# Shared Resistance, Phage-Receptor, and Biofilm Determinants in Antibiotic-Resistant *Pseudomonas aeruginosa*, *Escherichia coli*, and *Acinetobacter baumannii*: A Bibliometric and Gene-Network Analysis of Phage Cocktail Research

**DOI:** 10.3390/antibiotics15100950

**Published:** 2026-09-24

**Authors:** Wei Zhang, Peilin Zheng, Baohua He, Qi Li

**Affiliations:** 1 School of Public Health, Hebei Medical University, Shijiazhuang 050017, China; 25031100086@stu.hebmu.edu.cn (W.Z.); 23011050057@stu.hebmu.edu.cn (P.Z.); 2 Hebei Key Laboratory of Pathogens and Epidemiology of Infectious Diseases, Hebei Provincial Center for Disease Control and Prevention, Shijiazhuang 050021, China; 13582357836@163.com

**Keywords:** bacteriophage, phage cocktail, antimicrobial resistance, biofilm, *Acinetobacter baumannii*, bibliometric analysis, gene network

## Abstract

Background/Objectives: Antimicrobial resistance has renewed interest in bacteriophage cocktails as antibacterial and adjunctive agents, but the global research landscape and the bacterial determinants that constrain cocktail performance have not been mapped within a single framework. We quantified global phage-cocktail research and tested whether antibiotic-resistance, phage-receptor, and biofilm gene sets converge on shared genes in three Gram-negative pathogens representing three bacterial orders. Methods: The Web of Science Core Collection was searched on 5 July 2026 (query “phage” and “cocktail”). After deduplication and screening, 1598 articles and reviews (1992–2026) were analyzed with bibliometric workflows, citation-burst and co-citation analyses, and a focused China–United States comparison. Because these are the two leading producers and represent the dominant biocontrol and clinical research models, the comparison is deliberately restricted to them and is not presented as a global ranking. Resistance (R), phage receptor/defense (P), and biofilm (B) gene sets were curated for *Pseudomonas aeruginosa* PAO1, *Escherichia coli* K-12, and *Acinetobacter baumannii* ATCC 17978; overlaps were tested by one-sided hypergeometric tests with Benjamini–Hochberg correction and against size-matched control gene sets, followed by STRING protein–protein interaction and KEGG/GO enrichment. Orthologous conservation was assessed with KEGG Orthology in addition to gene-symbol matching. Results: Annual output grew from 2 records (1992–1996) to 873 (2022–2026; 54.6% of the corpus), led by the USA (322 records) and China (292). Phage-receptor–biofilm overlaps were strongly enriched in all three species (29, 7, and 28 genes; odds ratios 47.0, 8.1, and 31.7; all P ≤ 7.6 × 10^−5^), whereas resistance–phage-receptor overlaps were strongly enriched in PAO1 (18 genes; odds ratio 17.7) and K-12 (26 genes; odds ratio 30.2) but not in *A. baumannii* (9 genes; odds ratio 1.8; P = 0.088). Four unrelated control gene sets showed no enrichment in any species (odds ratios 0–1.9; P ≥ 0.42). Candidate networks formed alginate, Psl–Pel, lipopolysaccharide-modification, and SOS/porin modules; KEGG enrichment converged on biofilm formation and cationic antimicrobial peptide resistance; 14 of the 18 symbol-level conserved genes were corroborated by shared KEGG Orthology assignments. Conclusions: Phage-cocktail research has entered a phase of rapid growth, with hotspots converging on combination therapy and formulation. Biofilm-matrix synthesis and surface-appendage assembly form a transferable interface between phage adsorption and biofilm formation across three bacterial orders, whereas the coupling between antibiotic resistance and phage-receptor genes is lineage-restricted. Because the R, P, and B sets overlap by construction, these convergences are hypothesis-generating and require experimental validation before they can be used as design rules.

## 1. Introduction

Antimicrobial resistance (AMR) has become one of the most serious threats to global public health, eroding the efficacy of conventional antibiotics and driving the search for alternative and adjunctive antibacterial strategies [1,2]. Bacteriophages (phages), the viruses of bacteria, have experienced a remarkable renaissance over the past two decades as therapeutic, prophylactic, and biocontrol agents [2,3,4,5]. A century after their discovery, phages are again being deployed against multidrug-resistant infections, foodborne pathogens, and biofilm-associated disease, supported by advances in genomics, synthetic biology, and formulation science [2,5,6].

Single-phage preparations are limited by their narrow host ranges and by the rapid emergence of phage-resistant bacterial mutants. Phage cocktails—mixtures of phages with complementary receptor specificities and lytic properties—have therefore become a central strategy for broadening the spectrum of activity, delaying resistance, and achieving additive or synergistic killing [7,8]. Landmark clinical cases, such as the treatment of a disseminated *Acinetobacter baumannii* infection [9] and a drug-resistant *Mycobacterium abscessus* infection [10], the randomized PhagoBurn trial for *Pseudomonas aeruginosa* burn-wound infections [11], and advances in phage formulation and stabilization [12], illustrate both the promise and the remaining obstacles of cocktail-based approaches. In food safety, phage cocktails have repeatedly reduced contamination by *Escherichia coli* O157:H7 [13,14], *Salmonella* [15,16], *P. aeruginosa* biofilms [17], and *Campylobacter* in poultry [18].

The efficacy of any cocktail is ultimately constrained by bacterial determinants at three interacting levels: resistance mechanisms that protect the bacterium against antibiotics and phages alike, the surface receptors and defense systems that govern phage adsorption and replication, and the biofilm state that shields bacteria from both phages and antimicrobials [19,20,21,22,23,24]. Bacteria can lose or modify surface receptors, mask them with extracellular polymeric substances (EPS), or deploy CRISPR-Cas, restriction–modification, and SOS-based error-prone repair systems [19,21,22,23,24]. Many canonical antibiotic-resistance determinants—lipopolysaccharide (LPS) modification (*arn*/*pmr*), porin loss (*oprD*/*ompF*), and efflux—simultaneously alter phage receptor availability, while biofilm-matrix components such as alginate, Psl, and Pel are themselves phage-receptor determinants [19,20,25]. These dual roles are well established in the phage-biology literature and are therefore not, in themselves, a novel claim of the present work; what has not been quantified is whether the corresponding gene sets overlap more than expected by chance, whether such overlap survives ontology-based and specificity controls, and how it relates to the research landscape of the field. Biofilms are estimated to underlie the majority of chronic infections and are notoriously refractory to both antibiotics and phages [26]. Accordingly, rational cocktail design requires a systems-level view of the genes shared among resistance, phage receptor/defense, and biofilm networks [20,25,27,28,29,30,31].

Bibliometric analysis provides a quantitative map of the intellectual structure, collaboration networks, and research hotspots of a field [32,33,34,35]. Phage therapy has already been surveyed bibliometrically at the level of the whole field [36] and of specific subfields such as the phage–gut-microbiome interface [37], but these surveys treat phage cocktails as one keyword among many and remain at the level of research themes. Conversely, the mechanistic literature on phage receptors, bacterial defense, and biofilm matrices is extensive but has not been integrated with the observed research landscape [19,20,21,22,23,24,25]. The specific contribution of this study is therefore not the observation that resistance and phage-receptor functions can be encoded by the same genes—which is established [19,21,22,23,24]—but the quantitative, dual-species test of whether that overlap is statistically enriched, whether it is corroborated by ontology-based orthology checking and control-gene-set specificity testing, and how the resulting gene modules align with the bibliometric hotspots of the field. Here, we (i) mapped global research trends in phage-cocktail research from 1992 to 2026; (ii) benchmarked the scientific output of China and the United States, the two leading producers; and (iii) constructed and statistically tested resistance (R), phage receptor/defense (P), and biofilm (B) gene sets in the well-annotated model pathogens *P. aeruginosa* PAO1 [38] and *E. coli* K-12 [39], followed by protein–protein interaction (PPI) and KEGG/GO enrichment analyses [40,41,42], a KEGG Orthology cross-species comparison, and control-gene-set specificity analyses. Finally, we related the bibliometric hotspots to the enriched pathways and core candidate genes in a descriptive bridge map. The gene-level results are presented as computational and hypothesis-generating rather than as experimentally established design rules.

## 2. Results

### 2.1. Publication Output and Citation Trends

After deduplication and screening, 1598 articles and reviews published between 1992 and 2026 were retained. Output grew from 2 records in 1992–1996 and 3 in 1997–2001, through 16 (2002–2006) and 75 (2007–2011), to 166 (2012–2016), 463 (2017–2021), and 873 (2022–2026). These six five-year periods sum exactly to the full corpus of 1598 records, and the most recent period alone accounts for 54.6% of the total. Annual output peaked in 2025 (213 records), with 151 records already indexed in 2026 at the time of retrieval (Figure 1A). The corpus accumulated 57,396 citations (mean 35.9 per record; median 17), of which 131 records had at least 100 citations, while citations per paper declined slowly as annual output accelerated (Figure 1A). Record-level annual counts and citation totals are provided in Supplementary Table S7.

### 2.2. Countries, Institutions, and Collaboration Networks

Publications originated predominantly from the USA (322), China (292), India (134), the United Kingdom (109), and South Korea (82) (Figure 1B,C; Table 1). The USA also led in total citations (17,144), followed by China (7168) and the United Kingdom (5702) (Figure 1D). Among institutions, KU Leuven, the Egyptian Knowledge Bank, and Seoul National University were the most productive by author–institution count, whereas the University of California System, University of California San Diego, and KU Leuven led by total citations (Figure 1E and Figure 2A). International cooperation was dominated by China–USA–United Kingdom axes, with the strongest betweenness-centrality nodes in China, the United Kingdom, and Germany (Figure 2B). Complete country- and institution-level metrics, including centrality values, are given in Table 1.

### 2.3. Authors and Journals

The co-authorship network comprised 7568 author nodes connected by 42,812 collaborative edges. LAVIGNE R (34 records), PEREIRA C (24), ALMEIDA A (24), WAGEMANS J (21), and SULAKVELIDZE A (21) were the most productive authors, and LAVIGNE R (h = 24) and PIRNAY JP (h = 16) had the highest h-index (Figure 3A–C). By total citations, SCHOOLEY RT (2521 citations), PIRNAY JP (2368), LAVIGNE R (2267), and HATFULL GF (2086) ranked highest (Figure 3D; Table 2). Eigenvector-centrality analysis identified JABLONSKA J, ZAK N, and SBERRO-LIVNAT H as collaborative bridges, while ZHANG Y, KUTTER E, and BARR JJ ranked among the betweenness bridges (Table 2). Author-level indicators were computed from the Web of Science author fields and therefore inherit the initial-based author disambiguation of that database; homonymous authors may be merged, and this caveat applies to Figure 3B–D and Table 2.

The 1598 records were published in more than 400 journals. Frontiers in Microbiology (112 records; impact factor (IF) 5.8, JCR Q1) was the most productive journal, followed by Viruses (90; IF 3.8), Antibiotics (60; IF 5.5), Microbiology Spectrum (43; IF 4.1), and Applied and Environmental Microbiology (38; IF 4.2) (Figure 4A; Table 3). By total citations, Frontiers in Microbiology (4200) and Applied and Environmental Microbiology (3156) led, while Nature Medicine contributed the highest per-record citation impact (IF 52.5; Figure 4B). The dual-map overlay showed that phage-cocktail research mainly cites the molecular biology, genetics, microbiology, and immunology domains (Figure 4C).

### 2.4. Keywords and Knowledge Base

The most frequent keywords were bacteriophage (589), phage therapy (519), bacteriophages (390), biocontrol (292), and cocktail (251) (Figure 5B). The most frequent keyword pairs were bacteriophage + phage therapy (170 publications), bacteriophage + biocontrol (135), and bacteriophages + phage therapy (133) (Figure 5A). Burst detection identified the field’s accelerating core terms—phage therapy (strength 27.2, 2019–2026), bacteriophage (26.1, 2017–2026), bacteriophages (23.0, 2018–2026), antimicrobial resistance (17.6, 2020–2026), and biofilm (16.6, 2020–2026)—together with clinical, biocontrol, and formulation terms; the annual keyword heatmap shows their continuous growth (Figure 5C,D). Keyword co-occurrence clustering partitioned the field into six thematic clusters (108 nodes, 787 edges; Figure 5E).

The top 10 references by local citation counts (Table 4) were led by Labrie et al. [21], Chan et al. [8], Sulakvelidze et al. [43], Schooley et al. [9], and O’Flynn et al. [14], followed by Khan Mirzaei and Nilsson [44], Loc-Carrillo and Abedon [4], Kortright et al. [45], Dedrick et al. [10], and Bankevich et al. [46] (Figure 6A). The rankings by total and annual citations were led by methods and benchmarking references—Gapped BLAST [47], Trimmomatic [48], MEGA X [49], SPAdes [46], and Prokka [50]—followed by clinical and applied studies.

### 2.5. China–United States Comparison

Chinese-affiliated publications (n = 292) were concentrated in Beijing (51), Jiangsu (42), Guangdong (32), Shanghai (29), and Hubei (28), whereas US-affiliated publications (n = 322) were concentrated in Texas (25), Washington (17), New York (14), Florida (14), and Ohio (12) (Figure 7A,B). Chinese output grew steeply after 2018 and peaked in 2025 (51 records), while US output increased after 2017, with peaks of 36 records in 2021 and 33 records in 2024 (Figure 7C,D). The two corpora shared their most frequent terms, but with different relative emphasis: phage-cocktail and biofilm terms were proportionally more frequent among Chinese-affiliated publications, whereas *Pseudomonas aeruginosa* and bacteriophage-therapy terms were proportionally more frequent among US-affiliated publications (Figure 7E; Table 5). The journal impact factors of Chinese (median 4.75; mean 5.46) and US (median 4.65; mean 6.31) publications did not differ significantly (Mann–Whitney U = 47,097.5, P = 0.135; Welch’s t = −1.586, P = 0.114; Supplementary Figure S2). This comparison was deliberately restricted to the two leading producers, which together account for 38% of the corpus and exemplify the two dominant research models of the field (food-safety and biocontrol applications versus clinical translation); it is not a global ranking, and no inference is drawn about the relative standing of other countries. Full journal lists are provided in Supplementary Tables S3 and S4.

### 2.6. Gene-Set Overlap in P. aeruginosa PAO1 and E. coli K-12

In PAO1, the R (81 genes), P (105 genes), and B (73 genes) sets overlapped strongly for R∩P (18 genes; P = 3.5 × 10^−15^; OR = 17.7) and P∩B (29 genes; P = 1.5 × 10^−32^; OR = 47.0), whereas R∩B was limited to one gene (*rpoS*) and did not reach significance (P = 0.66) (Figure 8A). In K-12, R∩P comprised 26 genes (P = 4.9 × 10^−25^; OR = 30.2), P∩B comprised seven flagellar/motility genes (P = 7.6 × 10^−5^; OR = 8.1), and R∩B was empty (Figure 8B). No gene belonged to all three sets in either species; thus, 48 PAO1 and 33 K-12 candidate genes belonged to at least two sets. To establish that this enrichment is specific rather than a generic property of large gene sets, the same one-sided hypergeometric test was applied to four unrelated control gene sets of comparable size (central metabolism, ribosome/translation, bacterial secretion system, and purine metabolism). None of the twenty-four control contrasts was significant (OR 0–1.9; P = 0.42–1.00; Supplementary Table S9), indicating that the excess overlap is confined to the resistance, phage-receptor, and biofilm categories. A third species from a different order was analyzed separately as an independent validation (Section 2.11).

### 2.7. Protein–Protein Interaction Network of Candidate Genes

The PAO1 candidate network comprised 46 nodes and 227 edges at a combined-score threshold of 0.400 (45 nodes and 146 edges at ≥0.700) and resolved into four modules: alginate biosynthesis, Psl–Pel exopolysaccharide biosynthesis, LPS modification (Arn–PhoPQ), and SOS/porin functions (Figure 8C). Composite centrality ranking identified *pslA* (degree 26; betweenness 287.3; Z = 12.32), *algD* (20; 141.0; 7.07), *alg8* (17; 91.3; 4.52), *algU* (14; 77.8; 3.44), *pslD* (15; 30.1; 2.49), and *arnA* (12; 67.1; 2.26) as the network hubs. This ranking should be read with an important caveat: because the networks were constructed from members of known pathways, their known regulators are expected to emerge as hubs, and the analysis therefore recovers documented pathway architecture rather than identifying previously unrecognized regulators. Two SOS genes (*umuC*/*umuD*) were not represented in the STRING PAO1 network but were retained in the gene-set analysis.

### 2.8. Enrichment of Candidate Genes in Biofilm and CAMP-Resistance Pathways

Functional enrichment of the PAO1 candidates returned significant terms, including lipid A biosynthesis (7 genes; FDR = 4.6 × 10^−8^), lipopolysaccharide biosynthesis (7 genes; FDR = 7.8 × 10^−7^), alginate biosynthesis (6 genes; FDR = 4.1 × 10^−6^), antibiotic resistance (4 genes; FDR = 2.8 × 10^−3^), SOS response (3 genes; FDR = 4.3 × 10^−3^), and glycosyltransferase activity (4 genes; FDR = 6.0 × 10^−3^) (Figure 8D). KEGG enrichment in PAO1 converged on biofilm formation—*P. aeruginosa* (16 genes; FDR = 9.3 × 10^−14^), cationic antimicrobial peptide (CAMP) resistance (10 genes; FDR = 1.2 × 10^−11^), amino sugar and nucleotide sugar metabolism (5 genes; FDR = 8.0 × 10^−4^), two-component system (9 genes; FDR = 1.7 × 10^−3^), and fructose and mannose metabolism (3 genes; FDR = 1.7 × 10^−2^) (Figure 9A). In K-12, the top pathways were CAMP resistance (14 genes; FDR = 1.0 × 10^−17^), two-component system (12 genes; FDR = 2.8 × 10^−7^), flagellar assembly (7 genes; FDR = 1.2 × 10^−6^), lipopolysaccharide biosynthesis (4 genes; FDR = 7.6 × 10^−3^), and amino sugar and nucleotide sugar metabolism (4 genes; FDR = 1.3 × 10^−2^). GO enrichment of the PAO1 candidates yielded 29 significant biological-process terms led by cellular polysaccharide biosynthetic process (28 genes; FDR = 1.9 × 10^−30^), extracellular polysaccharide biosynthetic process (16 genes; FDR = 4.6 × 10^−22^), and single-species biofilm formation (18 genes; FDR = 5.5 × 10^−20^) (Figure 9B). Because enrichment is computed against the curated input sets, these terms summarize the functional content of those sets and should not be read as independent evidence for the pathways concerned.

### 2.9. Cross-Species Conservation and Bridging Analysis

Cross-species conservation was assessed first by direct gene-symbol comparison and then by an ontology-based check. Symbol matching identified 18 candidate genes shared between PAO1 and K-12 (30 PAO1-specific and 15 K-12-specific candidates): the *arnA*–*arnF*/*arnT* LPS-modification operon, the *phoP*/*phoQ* and *pmrB* two-component regulators, the SOS module (*dinB*, *lexA*, *recA*, *sulA*, *umuC*, *umuD*), and the flagellar genes *fliC* and *flgE* (Figure 9C). Because symbol matching is a text-level operation that can produce spurious matches, these calls were re-tested against KEGG Orthology (KO) assignments. KO identifiers were available for 43 of the 48 PAO1 and 29 of the 33 K-12 candidate genes, and 14 KO identifiers were shared between the two candidate sets, corroborating 14 of the 18 symbol-level matches (77.8%; Supplementary Table S8). The four uncorroborated matches were *pmrB*, for which the K-12 entry carries no KO assignment, and *sulA*, *umuC*, and *umuD*, which have no KO assignment in PAO1. The bridge map linked the bibliometric hotspots of the field—antimicrobial resistance, biofilm, phage cocktail, phage resistance, and two-component signaling—to the significantly enriched pathways (biofilm formation, CAMP resistance, two-component system, LPS biosynthesis, and amino sugar metabolism) and to core candidate genes (*pslA*, *algD*/*algU*, *arnA*/*arnB*, *phoP*/*phoQ*, *oprD*/*oprF*, and *lexA*/*recA*) (Figure 9D). This bridge map is a descriptive visual synthesis of the preceding analyses; the keyword-to-pathway-to-gene links were not themselves submitted to a statistical test and should not be read as independently validated associations. Conservation of these modules across the two model species is consistent with, but does not by itself demonstrate, generalizability: the genes concerned are broadly conserved across Gram-negative bacteria irrespective of their resistance, receptor, or biofilm classification, so their joint presence in PAO1 and K-12 was expected a priori. Whether the overlaps themselves extend beyond these two model systems was therefore tested in a third species belonging to a different order (Section 2.11).

### 2.10. Sensitivity of the Overlap Signal to Pre-Existing Dual Annotation

Because several genes in the LPS-modification and exopolysaccharide-matrix modules are already described in the literature as serving both resistance/receptor and biofilm functions, part of the observed overlap is definitional. To quantify this contribution, the overlap analysis was repeated after removing every previously dual-annotated gene from the R, P, and B sets (36 genes in PAO1 and 16 in K-12; Supplementary Table S10). R∩P remained significantly enriched in both species (PAO1: 8 genes, OR = 11.3, P = 2.1 × 10^−6^; K-12: 10 genes, OR = 11.7, P = 1.3 × 10^−7^), and P∩B remained significant in K-12 (7 genes, OR = 9.7; P = 2.6 × 10^−5^) but was attenuated in PAO1 (3 genes, OR = 5.6, P = 0.020). The resistance–phage-receptor convergence is therefore only partly attributable to shared prior annotation, whereas the phage-receptor–biofilm convergence in PAO1 is largely driven by the exopolysaccharide-matrix genes: alginate, Psl, and Pel accounted for 22 of the 29 P∩B genes (75.9%). Composition of the full overlaps is summarized in Supplementary Table S10. The same dual-annotation sensitivity analysis, together with a stricter redefinition of the phage-receptor set, was also applied to the third species (Section 2.11).

### 2.11. Validation in a Third, Distantly Related Gram-Negative Species

To test whether the overlaps identified in the two model pathogens extend beyond the Pseudomonadales and Enterobacterales, the same pipeline was applied to *Acinetobacter baumannii* ATCC 17978 (taxonomy ID 400667; Moraxellales), a species of growing clinical importance whose resistance, surface-receptor, and biofilm repertoires differ substantially from those of *P. aeruginosa* and *E. coli*. Because this strain lacks a widely used gene-symbol namespace, its R, P, and B sets (96, 213, and 45 genes) were assembled from pre-computed functional annotations together with RefSeq product names, and all members are reported by locus tag with their gene symbols in Supplementary Table S11.

The results are shown in Figure 10 and compared with the two model species in Supplementary Table S12. The P∩B overlap was again strong and highly significant (28 genes; P = 8.4 × 10^−25^; q = 2.5 × 10^−24^; OR = 31.7; Figure 10A,B), and its composition was biologically coherent: 18 of the 28 genes encode fimbrial or type IV pilus assembly and adhesion functions, including the CsuA/BABCDE chaperone–usher operon (CsuA, CsuB, CsuA/B, CsuD, and CsuE), the pilin-like competence factors A1S_3165/A1S_3166, and the FimT and type IV fimbrial biogenesis proteins, while the remaining members comprise surface-polysaccharide precursor enzymes (UDP-N-acetylglucosamine 2-epimerase, UDP-3-O-acyl-N-acetylglucosamine deacetylase), the alginate biosynthesis regulator AlgU, and the biofilm-associated exopolyphosphatase. In contrast, the R∩P overlap did not reach significance in this species (9 genes; P = 0.088; q = 0.13; OR = 1.8), and the nine genes concerned were dominated by lipid A modification and DNA-repair functions rather than by the porin and efflux mechanisms that dominate the two model species; R∩B was again empty.

The third-species result was robust to the two analytical choices that most plausibly affect it (Figure 10C; Supplementary Table S12). First, excluding broad defense-response annotations and prophage-structural product names from the P set (reducing it from 213 to 185 genes) left the P∩B signal essentially unchanged (27 genes; P = 4.8 × 10^−25^; q = 1.5 × 10^−24^; OR = 34.0) and moved R∩P only to borderline significance (P = 0.043; q = 0.065; OR = 2.1). Second, removing the 49 genes of the LPS/O-antigen modification module strengthened P∩B further (26 genes; P = 1.1 × 10^−25^; q = 3.3 × 10^−25^; OR = 40.0) while weakening R∩P (P = 0.37; OR = 1.3). Both variants therefore preserve the P∩B conclusion and leave the R∩P conclusion unsupported. Finally, the four size-matched control gene sets again produced no overlap with R, P, or B (0 of 12 contrasts significant; OR = 0; P = 1.00; Supplementary Table S13), so the *A. baumannii* signals are not a generic property of these set sizes.

Taken together with the two model species, these data separate the two convergences. The phage-receptor–biofilm coupling is recovered in three species that belong to three different orders and that use structurally unrelated adhesins (Psl/Pel and alginate in *P. aeruginosa*, curli and cellulose in *E. coli*, and Csu pili and poly-β-1,6-N-acetylglucosamine in *A. baumannii*), which argues that it reflects a general feature of the phage–host interface rather than a shared annotation artefact. The resistance–phage-receptor coupling, by contrast, is strong in PAO1 and K-12 but absent in *A. baumannii*, and is therefore better described as lineage-restricted than as a general property of Gram-negative pathogens.

## 3. Discussion

This study provides an integrated bibliometric and gene-network dissection of phage-cocktail research, showing that a field dormant for two decades expanded rapidly after 2017 and that its topical center has shifted from proof-of-concept phage therapy and food biocontrol toward combination therapy, formulation, and delivery. At the gene level, a core of resistance–phage-receptor–biofilm determinants—LPS and EPS biogenesis, two-component signaling, and SOS repair—connects the bibliometric hotspots to candidate mechanistic targets. The bibliometric component is descriptive, and the gene-network component is computational; together they generate hypotheses rather than establish mechanism.

The growth trajectory mirrors the broader phage-therapy renaissance [1,2,5]. The inflection around 2017 coincides with high-profile clinical successes and personalized compassionate-use cases [9,10], the PhagoBurn randomized trial [11], and expanding regulatory frameworks, with engineered and personalized phages emerging as a distinct frontier [10,51]. The dominance of Frontiers in Microbiology, Viruses, and Antibiotics—all open-access microbiology journals—reflects both the community’s composition and its preference for rapid dissemination. The USA’s citation lead (17,144) reflects landmark studies [9,10,11], whereas China’s rapid output growth indicates accelerating investment in phage biocontrol and clinical applications. Although Chinese publications were concentrated in food-safety-oriented journals and US publications in clinical journals, the impact-factor distributions were statistically indistinguishable (P = 0.135), suggesting comparable venue quality rather than a qualitative gap.

The central biological finding is a decoupling of the two convergences that the analysis was designed to detect. The phage-receptor–biofilm overlap was strong and significant in every species examined—PAO1 (OR = 47.0), K-12 (OR = 8.1), and *A. baumannii* ATCC 17978 (OR = 31.7)—and it was strengthened, rather than weakened, when genes with pre-existing dual annotation were removed (Section 2.10 and Section 2.11). Because these three species belong to three different orders and rely on structurally unrelated adhesins and matrix polymers, this coupling is best interpreted as a general property of the phage–host interface: the surface appendages and exopolysaccharides that phages exploit as receptors are the same structures that constitute and stabilize the biofilm matrix, so biofilm-forming cells present a distinct and largely shared set of adsorption targets. The resistance–phage-receptor overlap, by contrast, was strong in PAO1 (OR = 17.7) and K-12 (OR = 30.2) and survived the removal of dual-annotated genes in both species, but did not reach significance in *A. baumannii* (OR = 1.8; P = 0.088) and remained unsupported under every alternative set definition tested (Section 2.11). This axis therefore appears lineage-restricted rather than universal. In *P. aeruginosa* and *E. coli*, the dominant resistance mechanisms—LPS/core oligosaccharide modification (*arn*/*pmr*, *pagP*, *eptA*/*eptB*), porin remodelling (*oprD*, *oprF*, *ompC*/*ompF*), and SOS-mediated mutagenesis (*dinB*, *umuC*/*umuD*, *recA*/*lexA*)—are simultaneously canonical resistance determinants and determinants of phage adsorption, replication, and resistance evolution [19,20,21,23,24,25], whereas the *A. baumannii* resistance repertoire is dominated by efflux (AdeABC/AdeIJK), carbapenemase expression, and penicillin-binding-protein alterations, which are not themselves phage-binding structures. KEGG and GO enrichment independently support the same reading: biofilm formation and CAMP resistance dominate the PAO1 signal, whereas CAMP resistance and two-component signaling dominate K-12. CAMP resistance remains an informative convergence point wherever it occurs, because the same LPS-modification systems that confer resistance to polymyxins and host-defense peptides also remodel the surface molecules that phages use as receptors [19,25,27]; the third-species result nevertheless indicates that this coupling cannot be assumed for a new pathogen and must be established species by species.

These results suggest several testable design hypotheses. First, cocktails should target multiple mechanistically independent receptors (LPS, porins, and pili/flagella) to minimize the probability of simultaneous resistance [7,8,15,19]. Second, the receptor–biofilm coupling implies that anti-biofilm strategies are not merely adjunctive: matrix-degrading enzymes, anti-biofilm agents, or phages carrying EPS-degrading depolymerases act on the same surface structures that mediate adsorption, and this coupling was replicated in all three species examined [17,20,26]. Third, because the resistance–phage-receptor overlap was confined to the two model species, cocktails for a new pathogen should not be designed on the assumption that its resistance profile predicts its phage-receptor repertoire; the two gene sets must be re-tested for that species before the assumption is used [20,25,27]. Finally, combination with antibiotics and immune effectors could exploit the SOS and two-component nodes that couple resistance and phage defense wherever that coupling exists [28,30,31]. These predictions are directly testable in isogenic mutant panels and, if confirmed, would provide a rational counterpart to empirical cocktail screening; at present they remain computational.

Several limitations should be acknowledged. The corpus is restricted to the Web of Science Core Collection and to the query “phage” and “cocktail”; publications using alternative terminology (phage mixtures, polyphage therapy) or databases (PubMed, Scopus) may be underrepresented, and citation windows are truncated for recent records. Author-level indicators inherit the initial-based author disambiguation of the source database. The gene sets were curated from the literature and pathway annotations rather than generated by an automated detector, and the R, P, and B sets share some definitional components (notably LPS and EPS genes); the overlap enrichment should therefore be interpreted as a hypothesis-generating convergence rather than as independent proof. Four safeguards were applied: cross-checking R against the Comprehensive Antibiotic Resistance Database; using an independent PPI score threshold; (i) specificity controls against four unrelated gene sets; (ii) a sensitivity analysis that removes dual-annotated genes; and (iii) validation in a third, more distantly related species. Together, these showed that the P∩B signal is robust to the removal of dual-annotated genes and to the choice of set definition in all three species, whereas the R∩P signal survives in PAO1 and K-12 but not in *A. baumannii*. In addition, 4 of the 18 symbol-level conserved genes could not be corroborated by KEGG Orthology, and the bridge map in Figure 9D was not statistically tested. The analysis remains computational, so the identified networks and pathways should be validated experimentally in isogenic mutants and biofilm models. Three limitations specific to the third-species analysis should also be noted. First, its R, P, and B sets were assembled from pre-computed functional annotations and RefSeq product names rather than from literature curation, so they are more reproducible but less literature-informed than the two model-species sets; the stringent variant reported in Section 2.11 addresses the most probable consequence of that difference. Second, the P set of *A. baumannii* is proportionally larger (213 of 3787 proteins) than those of the model species, and a minority of its P∩B members carry only broad or electronically transferred annotations. Third, all species examined, including the validation species, are well-annotated laboratory strains and all are Gram-negative; the same pipeline should be extended to clinical isolates and to Gram-positive pathogens before any general claim of transferability is made.

## 4. Conclusions

Phage-cocktail research has grown from a niche topic into a rapidly expanding, transnational field whose hotspots now center on combination therapy, formulation, and delivery. A cross-species gene-network analysis of *P. aeruginosa* PAO1, *E. coli* K-12, and *A. baumannii* ATCC 17978 indicates that the transferable mechanistic core of phage–host interaction lies in biofilm-matrix synthesis and surface-appendage assembly, whereas the overlap between antibiotic-resistance and phage-receptor genes is lineage-restricted and should not be assumed for a new pathogen. These overlaps were statistically enriched and specific relative to unrelated control gene sets, but they are also partly definitional and the analyses are computational. The convergences reported here should therefore be regarded as hypothesis-generating candidates for experimental testing rather than as validated design rules for phage cocktails against drug-resistant infections.

## 5. Materials and Methods

### 5.1. Search Strategy and Data Collection

A systematic literature search was performed in the Web of Science Core Collection (WoS CC) on 5 July 2026 using the all-fields query “phage” and “cocktail”. Full records including cited references were exported from all subscribed WoS CC indexes and merged. The search returned 1660 raw records. Records were deduplicated in three steps: removal of one exact duplicate by Web of Science accession number (UT), and removal of two title-level duplicates for which equivalent records existed in different databases (the record with the complete DOI was retained), yielding 1657 unique records. Titles, abstracts, and keywords were screened against the topic of phage/bacteriophage cocktails; 19 records were excluded because they did not concern phages, leaving 1638 phage-related records. The corpus was restricted to original research articles and reviews, yielding 1598 records published between 1992 and 2026 (Article, n = 1354; Review, n = 220; and 24 additional article/review subtype records). The complete screening pipeline and record-level details are provided in the Supplementary Materials.

### 5.2. Bibliometric and Visual Analyses

Annual publication counts, cumulative and average citation trajectories, country/region productivity, institutional productivity, author productivity, and journal productivity were computed from the 1598-record corpus. Institutions were parsed from the Affiliations field; countries/regions were attributed from author addresses, so multi-country publications contributed to more than one country. Co-authorship networks among authors, countries, and institutions were constructed with degree, betweenness, and eigenvector centrality [32,35,52]. Keyword analysis combined Author Keywords and Keywords Plus after normalization (uppercase, single-space, and hyphen/space variant merging). Reference co-citation analysis was based on the cited-reference lists; the 200 most frequently co-cited references were retained as nodes and co-citation counts ≥7 defined edges, followed by Louvain community detection [53]. Bibliographic coupling among the top 200 references was analyzed analogously. All analyses and visualisations were implemented in Python (NumPy, pandas, SciPy and Matplotlib; Section 5.10).

### 5.3. China–United States Comparative Analysis

Publications with at least one Chinese or US affiliation were identified from the Addresses field (China, n = 292; USA, n = 322). Regional/state distributions, annual output trends, keyword frequency comparisons, and journal distributions were generated for each country. Journal impact factors were obtained from the Journal Citation Reports (JCR) 2025 release (Section 5.10) and compared by the Mann–Whitney U test and Welch’s *t*-test after excluding records with missing impact-factor data (China, n = 274; USA, n = 321). Keywords shared by the two countries (498 after normalization) were classified into five thematic categories.

### 5.4. Construction of Resistance, Phage Receptor/Defense, and Biofilm Gene Sets

Three Gram-negative model pathogens with complete, high-quality genome annotations were selected: *Pseudomonas aeruginosa* PAO1 (NCBI taxonomy ID 208964; STRING v12 background proteome N = 5571) [38], *Escherichia coli* K-12 MG1655 (taxonomy ID 511145; N = 4140) [39], and *Acinetobacter baumannii* ATCC 17978 (taxonomy ID 400667; N = 3787) [54]. Background proteome sizes were taken as the number of proteins listed for each species in STRING v12 [42]. Three non-exclusive gene sets were assembled from curated annotations and the literature as follows. For the two model species, the sets were curated from the literature and KEGG pathway annotations; because *A. baumannii* lacks a widely used gene-symbol namespace, the same three categories were operationalized for that species through STRING v12 pre-computed functional terms (GO biological process and cellular component, UniProt keywords, and Pfam domains) and RefSeq product names, using rules that were fixed before any overlap test was run [40,42]. The R (resistance) set was compiled from the principal antibiotic-resistance mechanisms of the two species, including intrinsic β-lactamase induction, efflux, porin loss, and LPS modification, and was cross-checked against CARD resistance-mechanism families [55]. The P (phage receptor/defense) set was compiled from the specialized reviews on phage receptors and bacterial anti-phage defense systems [19,21,22,23,24], together with KEGG pathway annotations for the corresponding surface structures (LPS and core oligosaccharide biosynthesis, porins, flagella, and type IV pili) and restriction–modification and SOS systems [41]. The B (biofilm) set was compiled from the biofilm-matrix and biofilm-regulation literature [26] together with KEGG biofilm-formation pathway annotations [41]. Set sizes were R (PAO1 n = 81; K-12 n = 69; *A. baumannii* n = 96), P (PAO1 n = 105; K-12 n = 106; *A. baumannii* n = 213), and B (PAO1 n = 73; K-12 n = 42; *A. baumannii* n = 45). No automated receptor-prediction or defense-system-detection tool (for example, PHIST or DefenseFinder) was applied; the sets therefore reflect the curated literature and pathway annotations available at the time of analysis, and complete gene lists with per-gene membership are provided in Supplementary Tables S5, S6 and S11.

### 5.5. Overlap Analysis and Statistical Testing

Pairwise and triple intersections among the R, P, and B sets were computed for each species. Overlap significance was assessed with one-sided hypergeometric tests against the species-specific proteome background (PAO1, N = 5571; K-12, N = 4140; *A. baumannii*, N = 3787), with Benjamini–Hochberg correction (BH-FDR). Cross-product odds ratios (OR) were calculated for each pairwise intersection. Candidate genes were defined as genes belonging to at least two of the three sets (PAO1, n = 48; K-12, n = 33; *A. baumannii*, n = 37). Two additional analyses were performed. First, to test whether the observed enrichment is specific rather than a generic property of large gene sets, the same one-sided hypergeometric test was applied to four control gene sets of comparable size retrieved from KEGG for each species—central metabolism (glycolysis, TCA cycle, and oxidative phosphorylation), ribosome/translation, bacterial secretion system, and purine metabolism—each tested against R, P, and B. Second, a sensitivity analysis repeated the overlap tests after removing every gene with pre-existing dual annotation from all three sets; the dual-annotated genes were the LPS-modification module (arn operon, *phoP*/*phoQ*, *pmrB*, and, in K-12, *pmrA*, *mgrB*, *pagP*, *eptA*, *eptB*, and *lpxO*) and the exopolysaccharide-matrix module (alginate, Psl, and Pel genes and their regulators), removing 36 genes in PAO1 and 16 in K-12. Control gene sets of comparable size were retrieved from KEGG for all three species [41]. For *A. baumannii*, the three sets were constructed by applying a fixed rule set to the STRING v12 pre-computed functional terms and to RefSeq product names, so that the same three conceptual categories were reproduced without relying on a gene-symbol namespace; a stringent P variant excluding broad defense-response annotations and prophage-structural product names, and a further analysis removing the 49-gene LPS/O-antigen modification module, were run in addition.

### 5.6. Protein–Protein Interaction Network Construction

Candidate-gene PPI networks were retrieved from the STRING database v12.0 API [42] using species-specific network endpoints. The primary network used a combined score threshold of ≥0.400, and a sensitivity network was generated at ≥0.700. Modules were identified with the CNM greedy modularity algorithm [56]. Hub genes were ranked by composite Z-scores integrating degree, betweenness, and closeness centrality.

### 5.7. Functional and Pathway Enrichment Analysis

Enrichment analyses were performed with the STRING v12.0 enrichment API [42] against species-specific backgrounds: KEGG pathways [41], Gene Ontology biological process (BP), molecular function (MF), and cellular component (CC) terms [40], and UniProt Keywords, Pfam, and InterPro domains. Significance was defined as BH-FDR q < 0.05. Because bacteria lack a Disease Ontology annotation layer, UniProt Keywords, Pfam, and InterPro terms served as the phenotype/function layer.

### 5.8. Cross-Species Comparison and Bridging Analysis

Cross-species conservation was first assessed by direct gene-symbol intersection of the PAO1 and K-12 candidate sets, and then verified against KEGG Orthology (KO) assignments. Gene symbols were mapped to KEGG locus tags and KO identifiers through the KEGG REST API; a symbol-level match was considered corroborated when the two candidate genes shared at least one KO identifier. R/P/B membership was displayed as a heatmap. A three-layer bridge map connected bibliometric hotspot keywords to significantly enriched KEGG pathways and, in turn, to core candidate genes; all gene–pathway links were based on STRING enrichment gene lists (q < 0.05) rather than subjective curation. The bridge map is a descriptive synthesis for visualization and was not subjected to a separate statistical test. The R∩P and P∩B odds ratios were additionally compared across the three species, and the robustness variants of the *A. baumannii* sets were displayed alongside the pre-specified result (Figure 10).

### 5.9. Statistical Analysis

Group comparisons used the Mann–Whitney U test and Welch’s *t*-test (two-sided; significance at *p* < 0.05). Hypergeometric tests were one-sided with BH-FDR adjustment (q < 0.05) and were complemented by the control-gene-set specificity tests and the dual-annotation sensitivity analysis described in Section 5.5. Author-level indicators (publication counts, h-index, and total citations) were derived from the Web of Science author fields and inherit the initial-based author disambiguation of that database. Analyses were performed in Python 3.9.6 with NumPy 2.0.2, pandas 2.3.3, SciPy 1.13.1 and Matplotlib 3.9.4 (Section 5.10); gene-symbol-to-orthology mapping used the KEGG REST API. Figures were generated with WorkBuddy v5.3.8 (Hy3 visualization engine) and reviewed by the authors; the language-polishing tool DeepSeek-V4-Flash is disclosed in the Acknowledgments.

### 5.10. Software and Database Versions

Literature search and record retrieval were performed in the Web of Science Core Collection (Clarivate Analytics, Philadelphia, PA, USA) on 5 July 2026, using all subscribed indexes. Journal impact factors were taken from the Journal Citation Reports 2025 release (Clarivate Analytics, Philadelphia, PA, USA). All bibliometric indicators, co-authorship, co-occurrence and co-citation networks, graph metrics (degree, betweenness, closeness and eigenvector centrality) and greedy-modularity (CNM) community detection, as well as all statistical tests, were computed in Python 3.9.6 (Python Software Foundation, Beaverton, OR, USA) using NumPy 2.0.2, pandas 2.3.3, SciPy 1.13.1 and Matplotlib 3.9.4; the network algorithms were implemented directly in Python and no external graph library was used. Gene annotation and functional enrichment were retrieved through the STRING v12.0 application programming interface (STRING Consortium, Zurich, Switzerland, and Heidelberg, Germany); KEGG pathway, Gene Ontology, UniProt Keywords, Pfam and InterPro annotations were supplied by STRING v12.0 through its enrichment endpoint, so that the annotation releases are those bundled in STRING v12.0. Taxonomic identifiers and background proteome sizes were taken from NCBI Taxonomy (National Center for Biotechnology Information, Bethesda, MD, USA). Resistance genes were cross-checked against the CARD resistance-mechanism families (McMaster University, Hamilton, ON, Canada). Figures were generated with WorkBuddy v5.3.8 including its Hy3 visualization engine (Tencent, Shenzhen, China). Data and analysis code are archived in Zenodo (CERN, Geneva, Switzerland; https://doi.org/10.5281/zenodo.22052112).

## Figures and Tables

**Figure 1:**
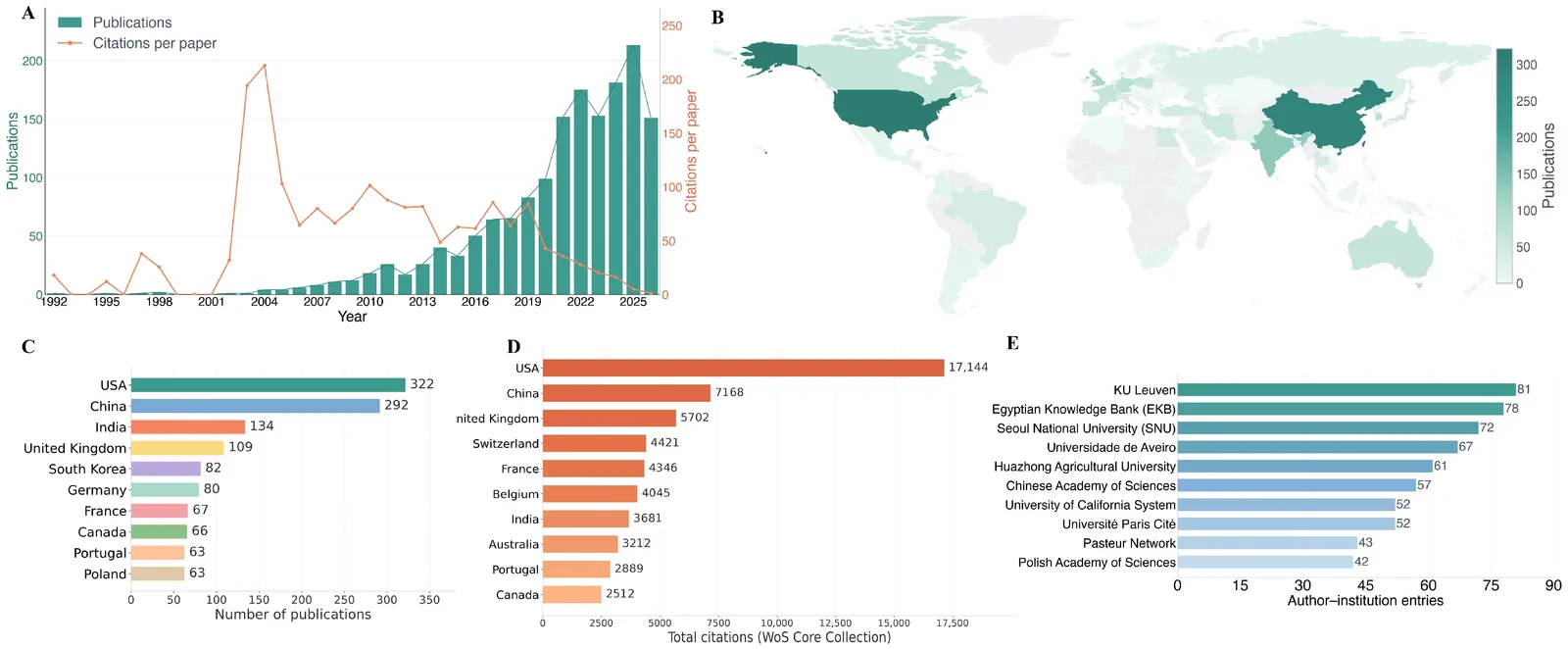
Global research profile of phage-cocktail publications (1992–2026). (**A**) Annual publication output and citations per paper; (**B**) geographic distribution of output; (**C**) top 10 countries/regions by output; (**D**) top 10 countries/regions by total citations; (**E**) top 10 institutions by author–institution count.

**Figure 2:**
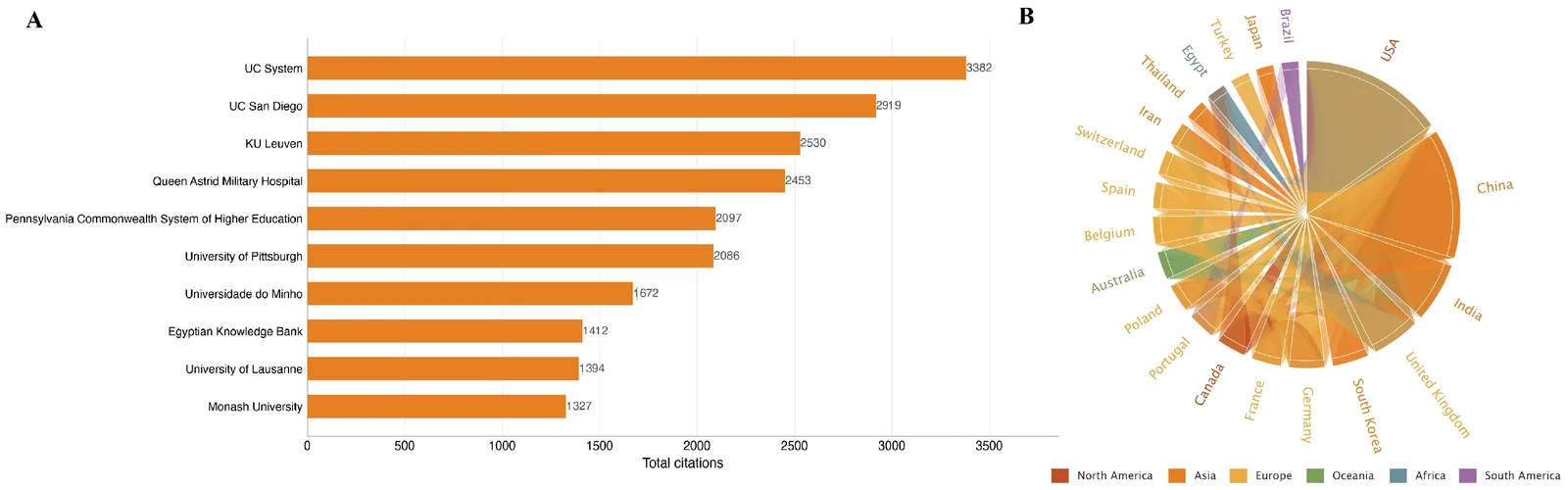
Institutional productivity and international cooperation. (**A**) Top 10 institutions by total citations; (**B**) chord diagram of international collaboration among the top 20 countries/regions.

**Figure 3:**
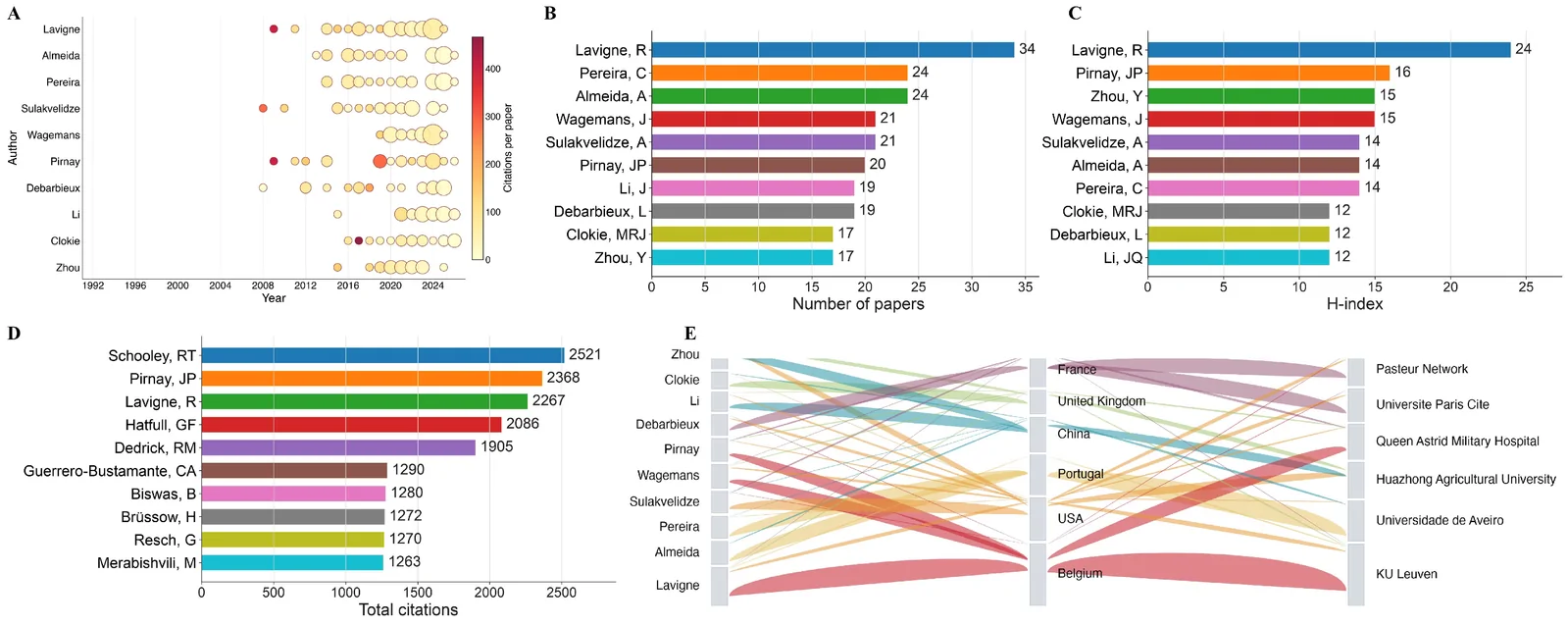
Author-level analysis of phage-cocktail research. (**A**) Bubble chart of the top 10 productive authors; (**B**–**D**) horizontal bar charts of the top 10 authors by number of papers, h-index, and total citations, respectively; (**E**) author–country–institution Sankey diagram. In (**A**), bubble size denotes the number of papers published by that author in a given year and bubble colour denotes citations per paper (colour bar); in (**B**–**D**), bars are coloured by author to allow tracking of the same author across panels; in (**E**), ribbon colour denotes country/region.

**Figure 4:**
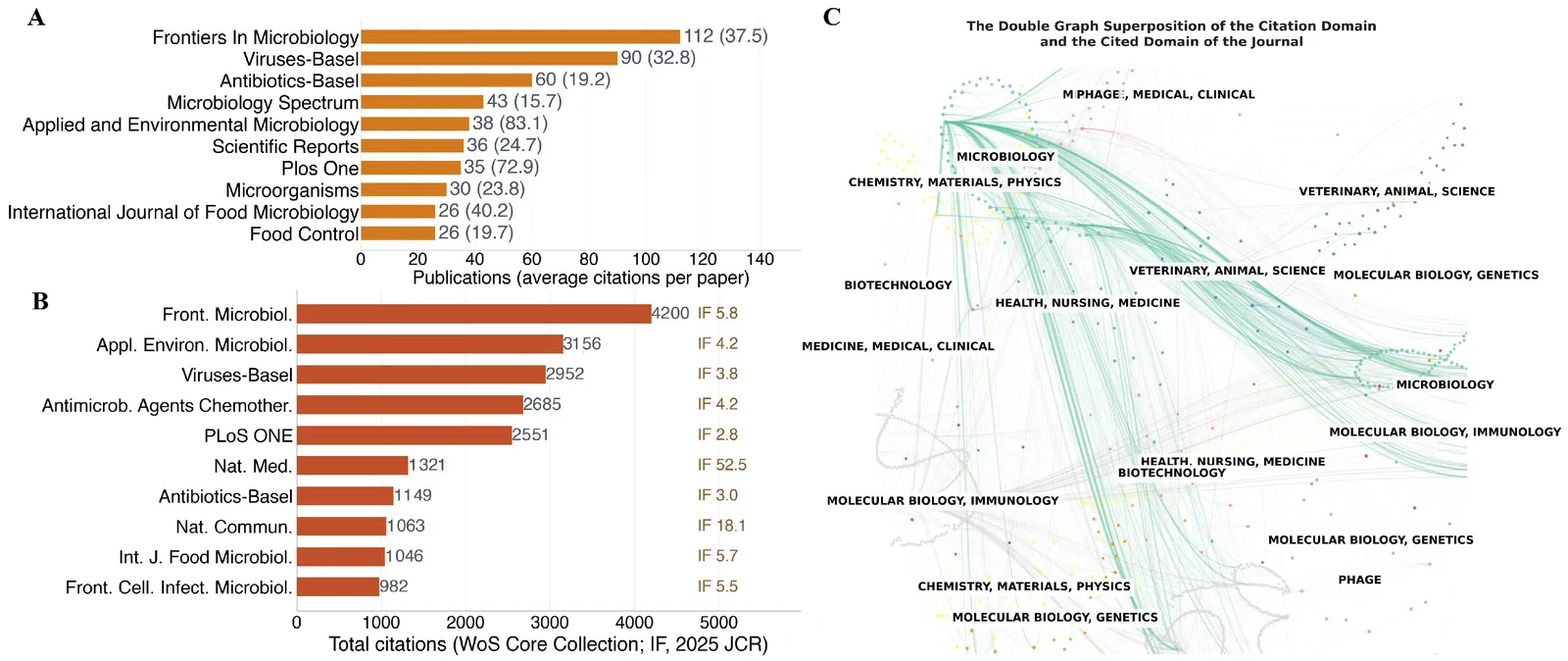
Journal-level analysis. (**A**) Top 10 journals by number of publications; (**B**) top 10 journals by total citations with the 2025 JCR impact factor; (**C**) dual-map overlay of citing and cited journal domains. Bars in (**A**,**B**) use a single colour, so colour carries no additional information. In the dual-map overlay (**C**), node colour denotes the journal cluster, and the curved lines trace citation links from the citing journal domain to the cited journal domain; label size scales with the number of journals in that domain.

**Figure 5:**
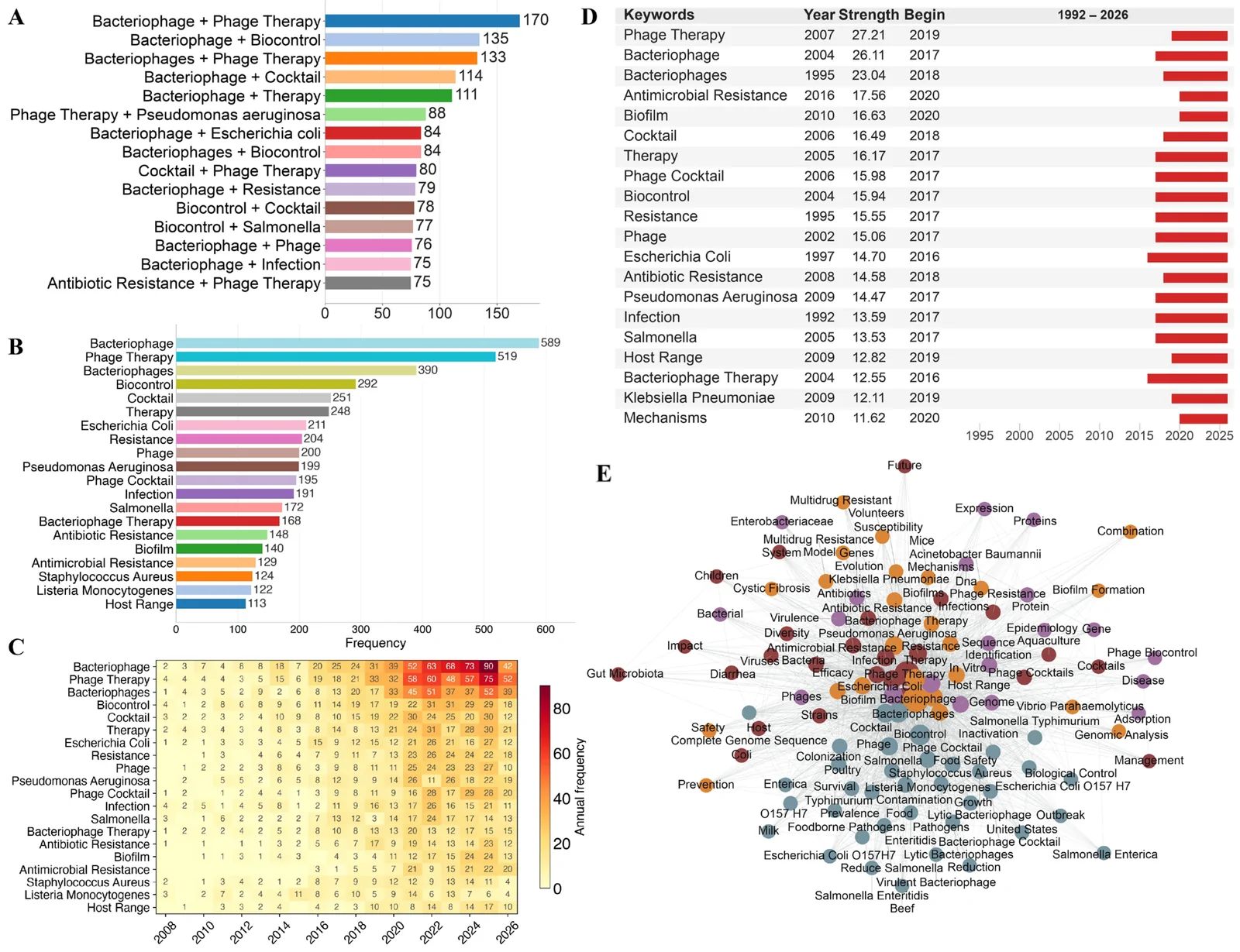
Keyword analysis of phage-cocktail research. (**A**) Most frequent keyword co-occurrence pairs; (**B**) ranked keyword frequencies; (**C**) annual keyword-frequency heatmap; (**D**) strongest citation bursts; (**E**) keyword co-occurrence clusters. Bars in (**A**,**B**) are coloured by keyword, with the same colour used for a given keyword in both panels; in the heatmap (**C**) colour encodes the annual keyword frequency (colour bar); in (**D**) red bars mark the detected burst interval for each keyword over 1992–2026; in (**E**) node colour denotes the keyword co-occurrence cluster and node size the keyword frequency.

**Figure 6:**
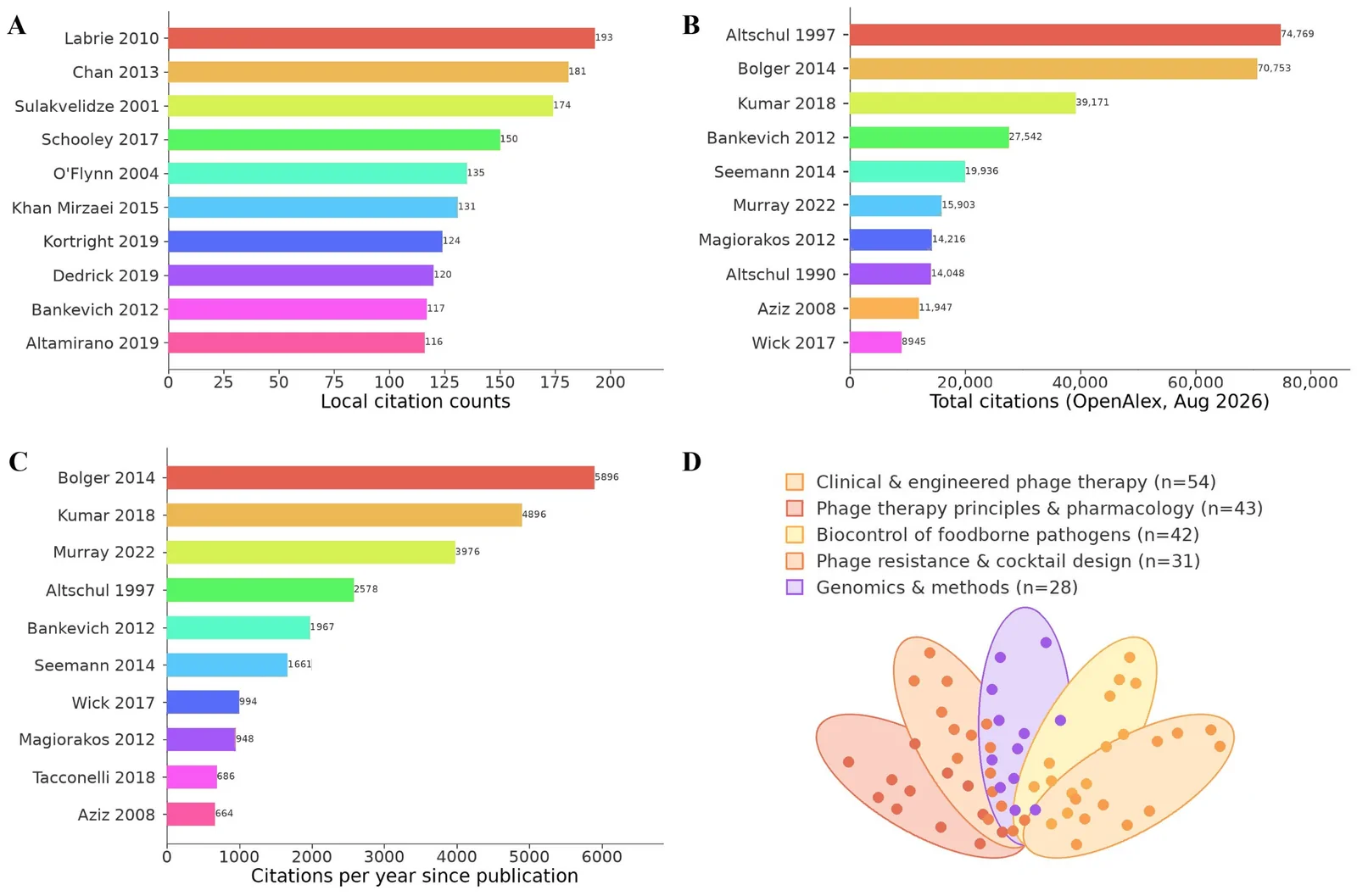
Reference-level citation analyses. (**A**) Top 10 references by local citations; (**B**) top 10 references by total citations; (**C**) top 10 references by annual citations; (**D**) bibliographic coupling network of the top 200 references.

**Figure 7:**
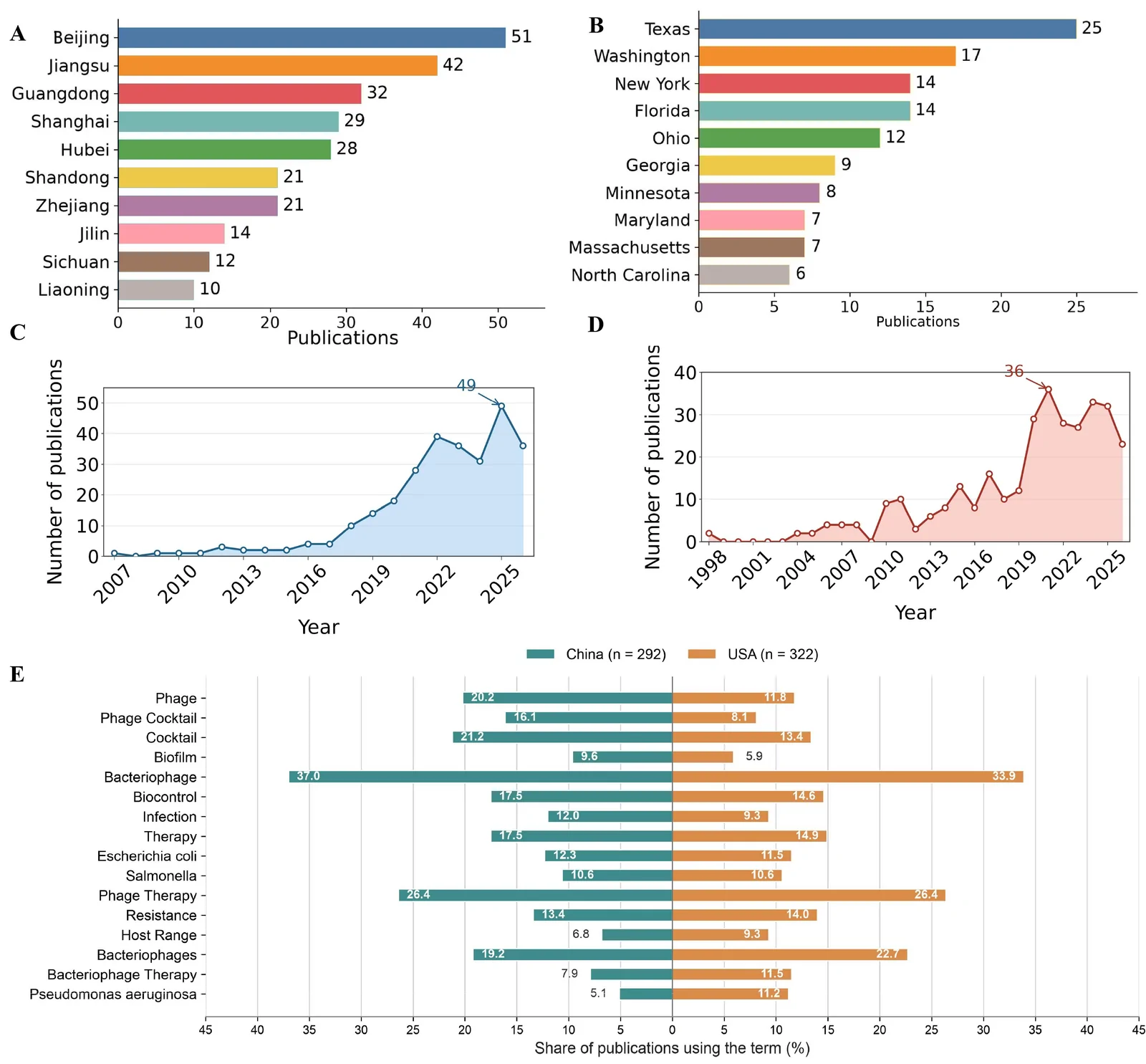
China–United States comparative analysis. (**A**,**B**) Top provinces/states of Chinese- and US-affiliated publications; (**C**,**D**) annual output trends; (**E**) diverging bar chart comparing the share of Chinese- and US-affiliated publications that use each of the 16 most frequent shared keywords.

**Figure 8:**
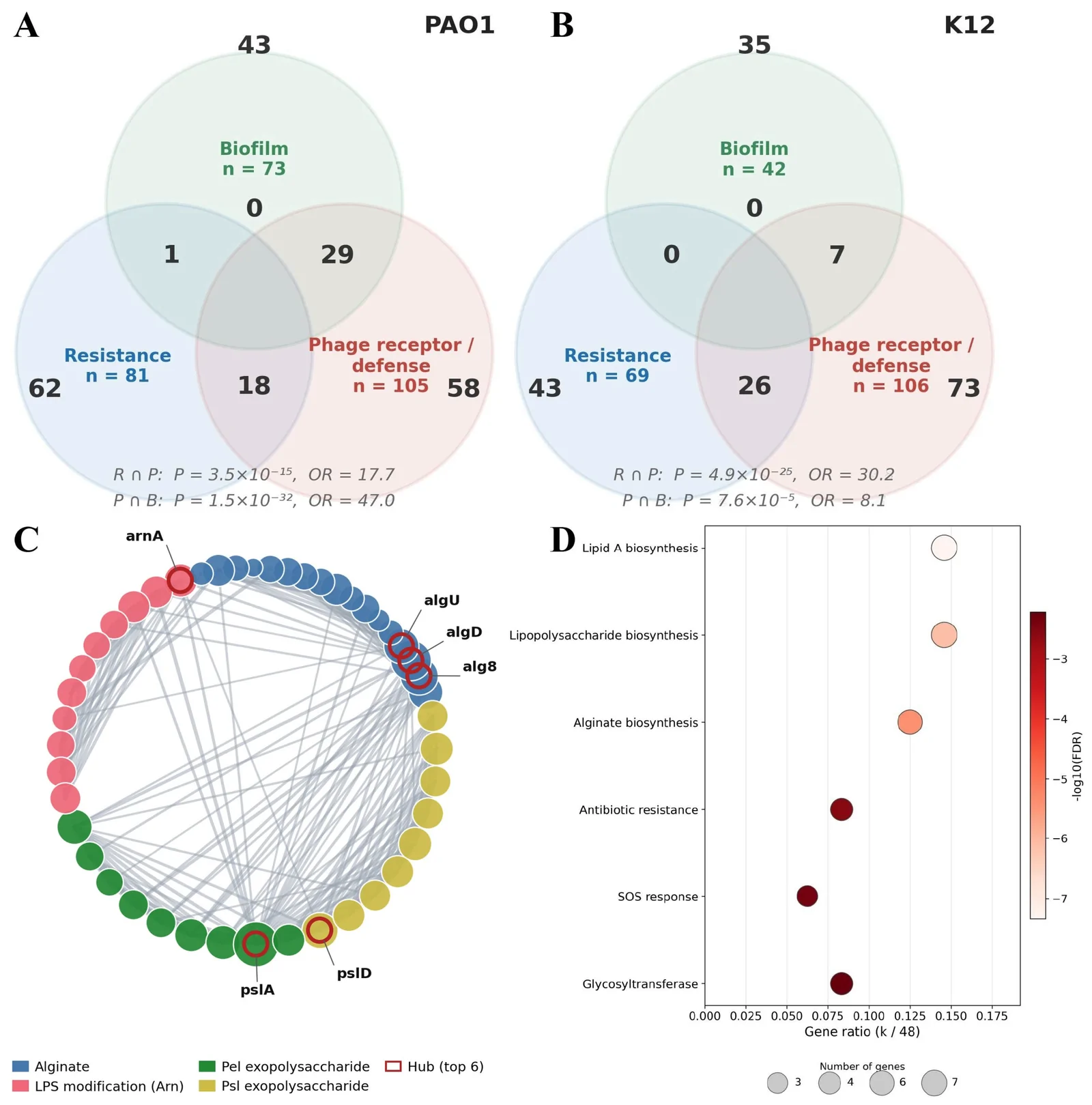
Gene-set and network analysis of candidate genes. (**A**,**B**) Venn diagrams of resistance (R), phage receptor/defense (P), and biofilm (B) sets; (**C**) STRING protein–protein interaction network of 48 PAO1 candidate genes; (**D**) functional enrichment.

**Figure 9:**
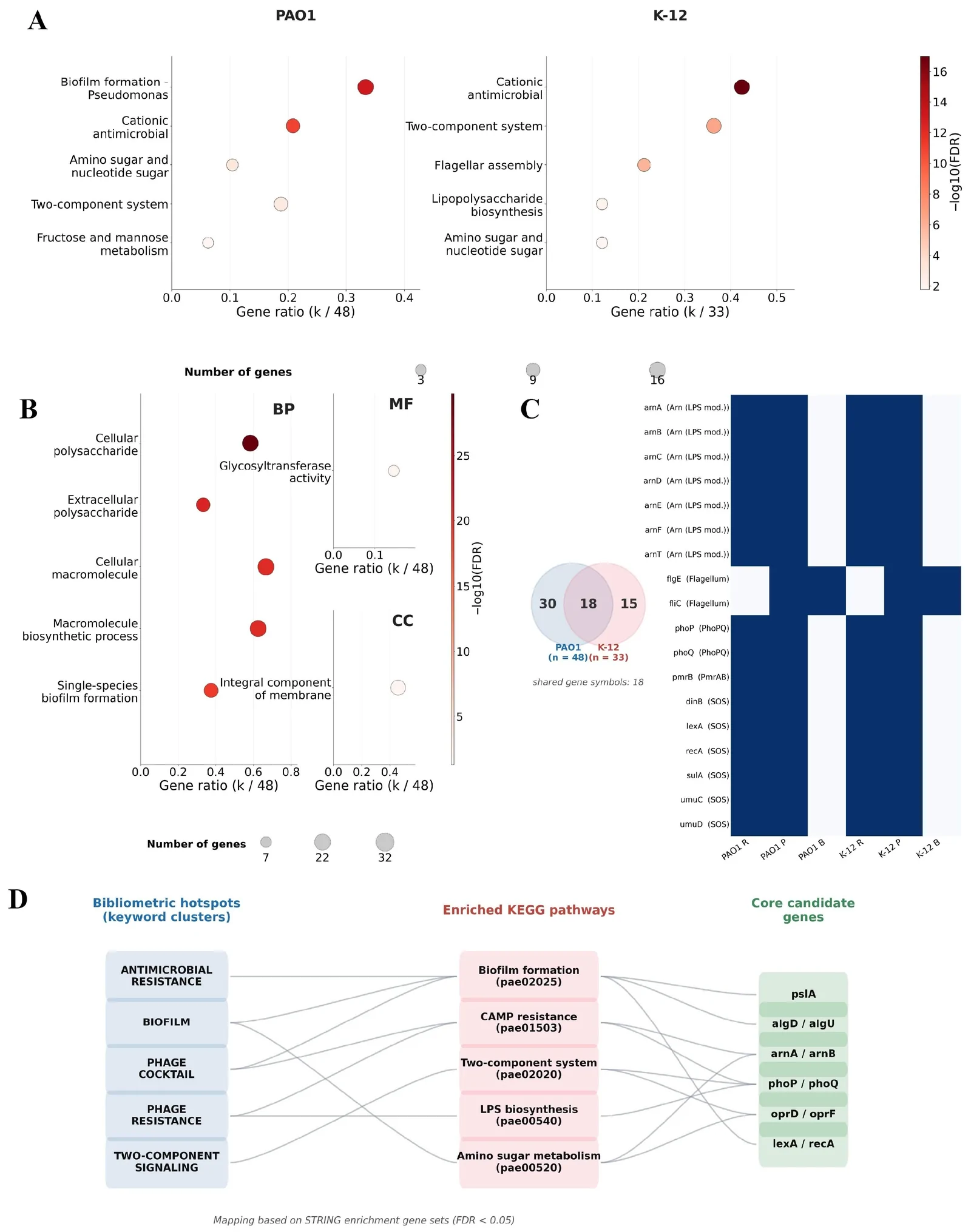
Pathway and mechanism-level analysis. (**A**) KEGG pathway enrichment; (**B**) GO enrichment; (**C**) cross-species comparison and R/P/B membership heatmap; (**D**) descriptive bridge map linking bibliometric hotspots, enriched pathways, and core candidate genes (visual synthesis only; links were not statistically tested).

**Figure 10:**
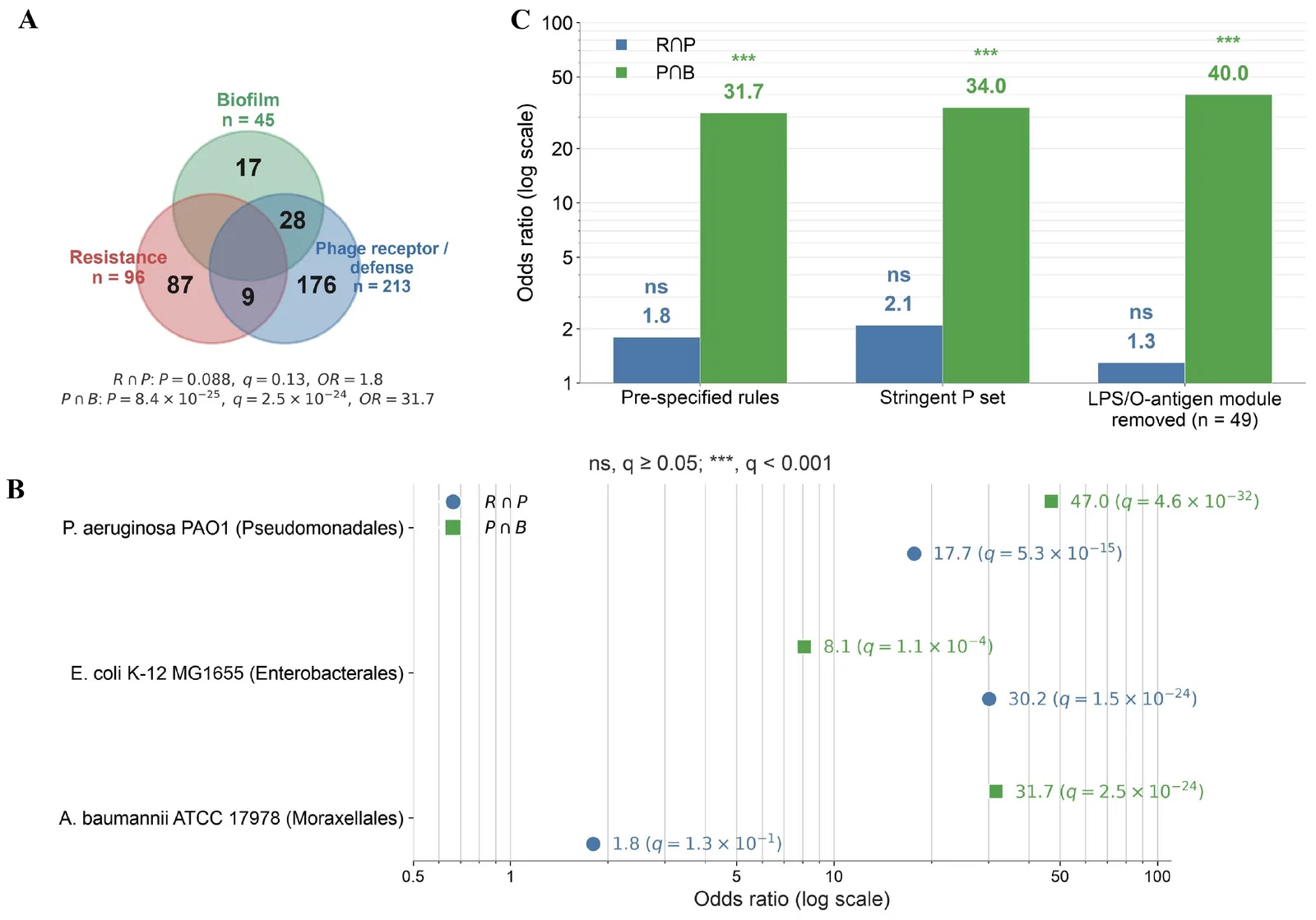
Third-species validation in *Acinetobacter baumannii* ATCC 17978. (**A**) Venn diagram of the resistance (R), phage receptor/defense (P), and biofilm (B) gene sets; (**B**) cross-species comparison of R∩P and P∩B odds ratios with Benjamini–Hochberg q values; (**C**) robustness of the *A. baumannii* overlaps under the pre-specified rules, a stringent P-set definition, and removal of the LPS/O-antigen modification module. Significance was assessed throughout on Benjamini–Hochberg adjusted q values from one-sided hypergeometric tests: ns, q ≥ 0.05; ***, q < 0.001.

**Table 1:** Cooperation-network centrality of countries/regions and institutions in the final 1598-record corpus.

Country	Percentage	Centrality	Organization	Percentage	Centrality
USA	20%	0.231	KU Leuven	1.3%	0.084
China	18%	0.116	Egyptian Knowledge Bank (EKB)	1.2%	0.069
India	8%	0.053	Seoul National University (SNU)	1.1%	0.018
United Kingdom	7%	0.114	Universidade de Aveiro	1.0%	0.012
South Korea	5%	0.007	Huazhong Agricultural University	0.9%	0.020
Germany	5%	0.075	Chinese Academy of Sciences	0.9%	0.093
France	4%	0.036	University of California System	0.8%	0.054
Canada	4%	0.025	Université Paris Cité	0.8%	0.016
Portugal	4%	0.034	Pasteur Network	0.7%	0.067
Poland	4%	0.004	Polish Academy of Sciences	0.7%	0.013

**Table 2:** Top 10 co-authors and network-central authors in the co-authorship network.

Rank	Co-Author	Papers	Eigenvector-Central Author	Centrality	Betweenness-Bridge Author	Centrality
1	LAVIGNE R	34	JABLONSKA J	0.173	LAVIGNE R	0.069
2	PEREIRA C	24	ZAK N	0.172	DEBARBIEUX L	0.041
3	ALMEIDA A	24	SBERRO-LIVNAT H	0.172	PIRNAY JP	0.037
4	WAGEMANS J	21	NICENBOIM J	0.172	LI J	0.036
5	SULAKVELIDZE A	21	GOLEMBO M	0.172	ZHANG Y	0.035
6	PIRNAY JP	20	MORDOCH R	0.172	KUTTER E	0.026
7	LI J	19	NAVOK S	0.172	CLOKIE MRJ	0.026
8	DEBARBIEUX L	19	BASSAN M	0.172	LI ZW	0.025
9	CLOKIE MRJ	17	WEINER I	0.134	BARR JJ	0.025
10	ZHOU Y	17	OLSHINA M	0.134	KUMAR A	0.022

**Table 3:** Impact factors of the top 10 journals by number of publications. IF (2025), 2025 JCR impact factor (released June 2026); JCR, JCR quartile.

Journal	Publications	Percentage	IF (2025)	JCR
Frontiers in Microbiology	112	7%	5.8	Q1
Viruses	90	6%	3.8	Q2
Antibiotics	60	4%	5.5	Q1
Microbiology Spectrum	43	3%	4.1	Q2
Applied and Environmental Microbiology	38	2%	4.2	Q1
Scientific Reports	36	2%	4.9	Q1
PLoS ONE	35	2%	2.8	Q1
Microorganisms	30	2%	4.7	Q2
Food Control	26	2%	7.0	Q1
International Journal of Food Microbiology	26	2%	5.7	Q1

**Table 4:** Top 10 references by local citation counts within the 1598-record corpus. Ref, reference list number; IF (2025), 2025 JCR impact factor.

Ref	Rank	Title	First Author	Year	Journal	IF (2025)	Local Citations
[21]	1	Bacteriophage resistance mechanisms	Labrie SJ	2010	Nature Reviews Microbiology	104.6	193
[8]	2	Phage cocktails and the future of phage therapy	Chan BK	2013	Future Microbiology	2.5	181
[43]	3	Bacteriophage therapy	Sulakvelidze A	2001	Antimicrobial Agents and Chemotherapy	4.2	174
[9]	4	Development and use of personalized bacteriophage-based therapeutic cocktails to treat a patient with a disseminated resistant *Acinetobacter baumannii* infection	Schooley RT	2017	Antimicrobial Agents and Chemotherapy	4.2	150
[14]	5	Evaluation of a cocktail of three bacteriophages for biocontrol of *Escherichia coli* O157:H7	O’Flynn G	2004	Applied and Environmental Microbiology	4.2	134
[44]	6	Isolation of phages for phage therapy: a comparison of spot tests and efficiency of plating analyses for determination of host range and efficacy	Khan Mirzaei M	2015	PLoS ONE	2.8	131
[4]	7	Pros and cons of phage therapy	Loc-Carrillo C	2011	Bacteriophage	N/A	129
[45]	8	Phage therapy: a renewed approach to combat antibiotic-resistant bacteria	Kortright KE	2019	Cell Host & Microbe	23.2	124
[10]	9	Engineered bacteriophages for treatment of a patient with a disseminated drug-resistant *Mycobacterium abscessus*	Dedrick RM	2019	Nature Medicine	52.5	120
[46]	10	SPAdes: a new genome assembly algorithm and its applications to single-cell sequencing	Bankevich A	2012	Journal of Computational Biology	1.6	117

**Table 5:** Keywords shared by China and the United States, reclassified into five thematic categories (top 25 per category shown).

Diseases and Clinical Conditions	Resistance and Pathophysiology	Phage and Bacterial Agents	Foods, Models, and Experimental Matrices	Molecules, Pathways, and Formulation
INFECTION	RESISTANCE	BACTERIOPHAGE	FOOD	SEQUENCE
INFECTIONS	BIOFILM	BACTERIOPHAGES	FOOD SAFETY	ANTIBIOTICS
DISEASE	BIOFILMS	BACTERIOPHAGE THERAPY	AQUACULTURE	ENDOLYSIN
DIARRHEA	HOST-RANGE	PHAGE	MILK	LIPOPOLYSACCHARIDE
PNEUMONIA	PHAGE RESISTANCE	PHAGES	BEEF	DELIVERY
MASTITIS	VIRULENCE	PHAGE THERAPY	GROUND-BEEF	ALIGNMENT
COVID-19	MECHANISMS	COCKTAIL	POULTRY	ENZYMES
COLITIS	CONTAMINATION	PHAGE COCKTAIL	LETTUCE	SYSTEM
BACTEREMIA	EVOLUTION	PHAGE COCKTAILS	MEAT	PERFORMANCE
WOUND	EFFICACY	SALMONELLA	CHICKEN SKIN	MANAGEMENT
SEPSIS	DIVERSITY	ESCHERICHIA-COLI	CHICKENS	TEMPERATURE
CANCER	INACTIVATION	LISTERIA-MONOCYTOGENES	CATTLE	GENOMICS
TUBERCULOSIS	SAFETY	TYPHIMURIUM	PIGS	QUALITY
SKIN	REDUCTION	STAPHYLOCOCCUS-AUREUS	FISH	HOLIN
CYSTIC FIBROSIS	LYSIS	PSEUDOMONAS AERUGINOSA	VEGETABLES	POLYSACCHARIDE
BURN	COLONIZATION	PSEUDOMONAS-AERUGINOSA	ENVIRONMENT	AGENT
AUREUS	GROWTH	BACTERIA	MICE	DEPOLYMERASE
OUTBREAK	BIOCONTROL	STRAINS	ZEBRAFISH	IMMUNE-SYSTEM
PREVALENCE	PHAGE-HOST INTERACTIONS	VIRUSES	CHILDREN	IMPACT
EPIDEMIOLOGY	GENE-EXPRESSION	ENTERICA	MODEL	STABILITY
TRANS-ETHNIC STUDY	SINGLE	PARAHAEMOLYTICUS	INTESTINE	FORMULATION
VACCINES	PATHOGENICITY	GENOME	TOOL	OPTIMIZATION
PATHOGENESIS	ANTIBACTERIAL	GENES	DATABASE	PRODUCTS
MULTIDRUG RESISTANCE	IN-VITRO	GENE	PREDICTION	FUTURE
ANTIBIOTIC-RESISTANCE	ENGINEERED PHAGE	DNA	IN VITRO	TAILSPIKE PROTEIN

## Data Availability

The derived data supporting the reported results are included in the article and Supplementary Materials. The curated gene sets, derived data tables, and analysis scripts are openly available in Zenodo at https://doi.org/10.5281/zenodo.22052112 (all-versions DOI, always resolving to the latest release; version 2, containing the specificity-control, orthology, dual-annotation, and third-species validation analyses added during revision; accessed on 13 September 2026).

## Supplementary Materials

The following supporting information can be downloaded at https://www.mdpi.com/article/10.3390/antibiotics15100950/s1, Figure S1: Reference knowledge base of phage-cocktail research. (A) Top 10 references with the strongest citation bursts; (B) co-citation network of references (200 nodes, 2,909 edges); (C) Sankey diagram linking references, authors, and keywords; Figure S2: Journal impact factors of Chinese- and US-affiliated publications. Box-and-violin plot (China: median 4.75, mean 5.46; USA: median 4.65, mean 6.31; Mann–Whitney U = 47,097.5, P = 0.135); Table S1: The 48 PAO1 candidate genes and their membership in the resistance (R), phage receptor/defense (P), and biofilm (B) gene sets (1, member; 0, non-member); Table S2: The 33 K-12 candidate genes and their membership in the resistance (R), phage receptor/defense (P), and biofilm (B) gene sets (1, member; 0, non-member); Table S3: Journals of publications affiliated with China (121 journals). Impact factors from the 2025 JCR release (released June 2026); Table S4: Journals of publications affiliated with the United States (133 journals). Impact factors from the 2025 JCR release (released June 2026); Table S5: Complete gene list for *Pseudomonas aeruginosa* PAO1: all 211 genes in the resistance (R), phage receptor/defense (P), and biofilm (B) gene sets, with per-gene membership (1, member; 0, non-member); Table S6: Complete gene list for *Escherichia coli* K-12 MG1655: all 184 genes in the resistance (R), phage receptor/defense (P), and biofilm (B) gene sets, with per-gene membership (1, member; 0, non-member); Table S7: Annual publication output and citations of the 1598-record corpus (1992–2026); Table S8: KEGG Orthology (KO) verification of the 48 PAO1 candidate genes. Gene symbols are italic. “Corroborated” indicates that at least one KO identifier is shared with a K-12 candidate gene; Table S9: Specificity control. Overlap of the resistance (R), phage receptor/defense (P), and biofilm (B) gene sets with four unrelated control gene sets of comparable size, tested by the same one-sided hypergeometric test against the same species background. Odds ratios (OR) and uncorrected P values are shown; n is the intersection size; Table S10: Sensitivity of the overlap enrichment to pre-existing dual annotation. Genes already described as dual-function in the prior literature (LPS-modification and exopolysaccharide-matrix modules) were removed from all three gene sets before the overlap tests were repeated (36 genes in PAO1, 16 in K-12); Table S11: Complete gene list for Acinetobacter baumannii ATCC 17978: all 317 genes in the resistance (R), phage receptor/defense (P), and biofilm (B) sets, with per-gene membership (1 = member, 0 = non-member). Locus tags follow the ATCC 17978 annotation (taxonomy ID 400667); gene symbols and product descriptions were taken from KEGG (organism code acb) and from RefSeq product names, because this strain has no widely used gene-symbol namespace. Set sizes: R, n = 96; P, n = 213; B, n = 45; Table S12: Cross-species summary of the R/P/B overlap analysis (upper block) and robustness variants for Acinetobacter baumannii ATCC 17978 (lower block). One-sided hypergeometric tests against the species-specific STRING v12 background; q = Benjamini–Hochberg adjusted P; Table S13: Specificity control for the Acinetobacter baumannii ATCC 17978 gene sets. Overlap of the resistance (R), phage receptor/defense (P), and biofilm (B) sets with four unrelated control gene sets of comparable size, tested by the same one-sided hypergeometric test against the same background (N = 3787). The second block reproduces the observed overlaps for direct comparison.
