# Supplementary material for: Shared Resistance, Phage-Receptor, and Biofilm Determinants in Antibiotic-Resistant Pseudomonas aeruginosa, Escherichia coli, and Acinetobacter baumannii: A Bibliometric and Gene-Network Analysis of Phage Cocktail Research
Source: Antibiotics (Basel). 2026 Sep 24;15(10):950. doi: 10.3390/antibiotics15100950 (PMC13700643; doi:10.3390/antibiotics15100950)
Supplement: Supplementary file 1 [file antibiotics-15-00950-s001.zip › antibiotics-4548169-supplementary.pdf]

Supplementary Materials

Shared Resistance, Phage-Receptor, and Biofilm Determinants in Antibiotic-Resistant *Pseudomonas aeruginosa*, *Escherichia coli*, and *Acinetobacter baumannii*: A Bibliometric and Gene-Network Analysis of Phage Cocktail Research

Supplementary Figures

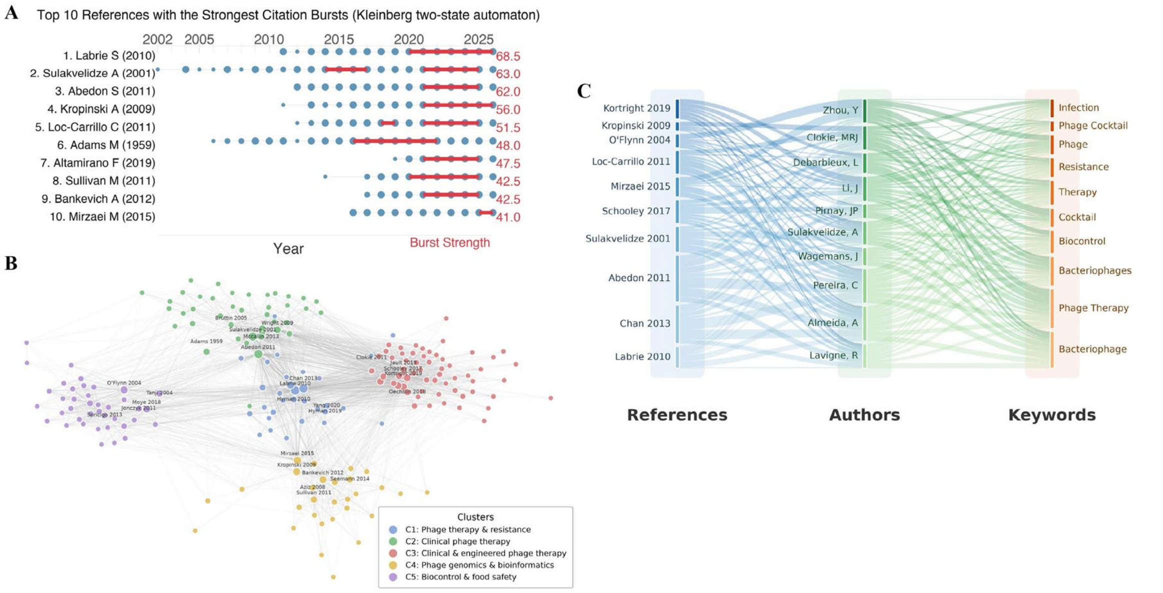

Figure S1. Reference knowledge base of phage-cocktail research. (A) Top 10 references with the strongest citation bursts; (B) co-citation network of references (200 nodes, 2,909 edges); (C) Sankey diagram linking references, authors, and keywords.

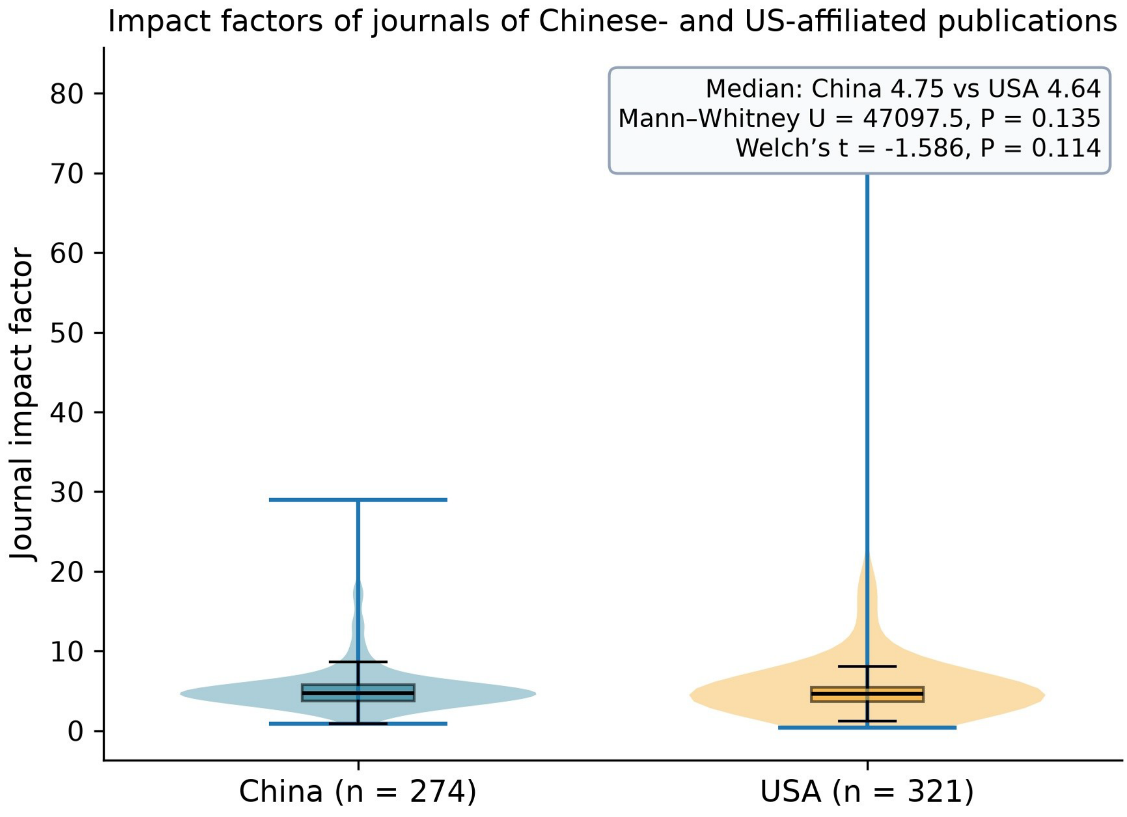

Figure S2. Journal impact factors of Chinese- and US-affiliated publications. Box-and-violin plot (China: median 4.75, mean 5.46; USA: median 4.65, mean 6.31; Mann–Whitney U = 47,097.5, P = 0.135).

Supplementary Tables

Table S1. The 48 PAO1 candidate genes and their membership in the resistance (R), phage receptor/defense (P), and biofilm (B) gene sets (1, member; 0, non-member).

Gene	R	P	B
alg44	0	1	1
alg8	0	1	1
algD	0	1	1
algU	0	1	1
arnA	1	1	0
arnB	1	1	0
arnC	1	1	0

Gene	R	P	B
arnD	1	1	0
arnE	1	1	0
arnF	1	1	0
arnT	1	1	0
dinB	1	1	0
flgE	0	1	1
fliC	0	1	1
lexA	1	1	0
mucA	0	1	1
mucB	0	1	1
mucC	0	1	1
mucD	0	1	1
oprD	1	1	0
oprF	1	1	0
pelA	0	1	1
pelB	0	1	1
pelC	0	1	1
pelD	0	1	1
pelE	0	1	1
pelF	0	1	1
pelG	0	1	1

Gene	R	P	B
phoP	1	1	0
phoQ	1	1	0
pilA	0	1	1
pmrB	1	1	0
pslA	0	1	1
pslD	0	1	1
pslE	0	1	1
pslF	0	1	1
pslI	0	1	1
pslJ	0	1	1
pslK	0	1	1
pslL	0	1	1
pslM	0	1	1
pslN	0	1	1
pslO	0	1	1
recA	1	1	0
rpoS	1	0	1
sulA	1	1	0
umuC	1	1	0
umuD	1	1	0

Table S2. The 33 K-12 candidate genes and their membership in the resistance (R), phage receptor/defense (P), and biofilm (B) gene sets (1, member; 0, non-member).

Gene	R	P	B
arnA	1	1	0
arnB	1	1	0
arnC	1	1	0
arnD	1	1	0
arnE	1	1	0
arnF	1	1	0
arnT	1	1	0
dinB	1	1	0
eptA	1	1	0
eptB	1	1	0
flgE	0	1	1
flhC	0	1	1
flhD	0	1	1
fliA	0	1	1
fliC	0	1	1
lexA	1	1	0
lpxO	1	1	0
mgrB	1	1	0
motA	0	1	1
motB	0	1	1

Gene	R	P	B
ompC	1	1	0
ompF	1	1	0
pagP	1	1	0
phoP	1	1	0
phoQ	1	1	0
pmrA	1	1	0
pmrB	1	1	0
recA	1	1	0
recF	1	1	0
recN	1	1	0
sulA	1	1	0
umuC	1	1	0
umuD	1	1	0

Table S3. Journals of publications affiliated with China (121 journals). Impact factors from the 2025 JCR release (released June 2026).

Rank	Journal	Publications	Percentage (%)	Impact Factor
1	Frontiers In Microbiology	30	10.3	5.800
2	Microorganisms	11	3.8	4.700
3	Microbiology Spectrum	11	3.8	4.100
4	Aquaculture	10	3.4	4.444
5	Microbial Pathogenesis	9	3.1	3.764

Rank	Journal	Publications	Percentage (%)	Impact Factor
6	Viruses	8	2.7	3.800
7	Food Control	7	2.4	7.000
8	Lwt-Food Science And Technology	7	2.4	5.882
9	Food Research International	7	2.4	8.039
10	Frontiers In Cellular And Infection Microbiology	6	2.1	5.599
11	International Journal Of Food Microbiology	6	2.1	5.700
12	Antibiotics	6	2.1	5.5
13	Food Microbiology	5	1.7	5.312
14	Applied And Environmental Microbiology	5	1.7	4.200
15	Plos One	5	1.7	2.800
16	Microbiological Research	4	1.4	6.674
17	Archives Of Virology	4	1.4	2.735
18	International Journal Of Antimicrobial Agents	4	1.4	10.290
19	Poultry Science	4	1.4	4.357
20	Virus Genes	4	1.4	1.667
21	Virus Research	4	1.4	4.950
22	Virologica Sinica	4	1.4	5.542
23	Biology-Basel	4	1.4	4.120
24	Journal Of Applied Microbiology	3	1.0	3.582
25	Applied Microbiology And Biotechnology	3	1.0	4.931
26	Virology Journal	3	1.0	4.752
27	Nature Communications	3	1.0	17.694

Rank	Journal	Publications	Percentage (%)	Impact Factor
28	Veterinary Microbiology	3	1.0	3.255
29	Bmc Microbiology	3	1.0	4.213
30	Virology	3	1.0	3.664
31	Quality Assurance And Safety Of Crops & Foods	2	0.7	3.899
32	Foodborne Pathogens And Disease	2	0.7	2.369
33	Current Research In Food Science	2	0.7	N/A
34	Emerging Microbes & Infections	2	0.7	13.057
35	Fish & Shellfish Immunology	2	0.7	4.607
36	Mbio	2	0.7	6.265
37	Frontiers In Veterinary Science	2	0.7	3.181
38	Current Microbiology	2	0.7	2.622
39	International Journal Of Molecular Sciences	2	0.7	6.208
40	Journal Of Food Protection	2	0.7	1.984
41	Journal Of Hazardous Materials	2	0.7	13.561
42	Journal Of Dairy Science	2	0.7	3.448
43	Infection And Drug Resistance	2	0.7	3.885
44	Food Bioscience	2	0.7	5.096
45	Msystems	2	0.7	6.207
46	Meat Science	1	0.3	6.958
47	Angewandte Chemie-International Edition	1	0.3	16.268
48	Cell Host & Microbe	1	0.3	23.2
49	Food & Function	1	0.3	6.065

Rank	Journal	Publications	Percentage (%)	Impact Factor
50	Folia Microbiologica	1	0.3	N/A
51	Aquaculture Reports	1	0.3	N/A
52	Current Pharmaceutical Design	1	0.3	3.072
53	Journal Of Cancer Research And Clinical Oncology	1	0.3	3.511
54	International Journal Of Food Science And Technology	1	0.3	3.164
55	Animal Feed Science And Technology	1	0.3	3.204
56	Biomedicines	1	0.3	N/A
57	Gut Microbes	1	0.3	12.220
58	Tissue Engineering And Regenerative Medicine	1	0.3	3.621
59	Chinese Medical Journal	1	0.3	5.877
60	Iscience	1	0.3	5.740
61	International Dairy Journal	1	0.3	N/A
62	Research In Veterinary Science	1	0.3	N/A
63	Archives Of Microbiology	1	0.3	2.780
64	Chemical Science	1	0.3	8.378
65	Fems Microbiology Letters	1	0.3	N/A
66	Environmental Microbiology	1	0.3	4.909
67	World Rabbit Science	1	0.3	0.843
68	Combinatorial Chemistry & High Throughput Screening	1	0.3	1.580
69	Infection	1	0.3	7.647
70	Future Microbiology	1	0.3	2.5

Rank	Journal	Publications	Percentage (%)	Impact Factor
71	Applied Biochemistry And Biotechnology	1	0.3	2.916
72	International Journal Of Infectious Diseases	1	0.3	8.616
73	Materials Today Bio	1	0.3	8.084
74	Frontiers In Immunology	1	0.3	7.251
75	Gels	1	0.3	N/A
76	Pesticide Biochemistry And Physiology	1	0.3	4.701
77	Plos Pathogens	1	0.3	6.651
78	Molecular Genetics And Genomics	1	0.3	2.873
79	Biocontrol Science And Technology	1	0.3	1.348
80	Pharmaceutics	1	0.3	N/A
81	Acs Nano	1	0.3	17.054
82	Frontiers In Bioscience-Landmark	1	0.3	2.849
83	Journal Of Infectious Diseases	1	0.3	6.145
84	Canadian Journal Of Microbiology	1	0.3	2.750
85	Science Of The Total Environment	1	0.3	10.753
86	Virus Evolution	1	0.3	5.824
87	Microbial Drug Resistance	1	0.3	2.620
88	Ecotoxicology And Environmental Safety	1	0.3	6.753
89	Parasites & Vectors	1	0.3	3.164
90	Pest Management Science	1	0.3	4.016
91	International Microbiology	1	0.3	3.094
92	International Journal Of Pharmaceutics	1	0.3	5.759

Rank	Journal	Publications	Percentage (%)	Impact Factor
93	Frontiers In Plant Science	1	0.3	N/A
94	Environmental Research	1	0.3	8.257
95	Ebiomedicine	1	0.3	11.017
96	Pathogens	1	0.3	3.640
97	Advanced Materials	1	0.3	28.488
98	Sensors And Actuators B-Chemical	1	0.3	8.279
99	Analytica Chimica Acta	1	0.3	6.083
100	Letters In Applied Microbiology	1	0.3	2.309
101	Veterinary Research	1	0.3	4.413
102	Microchemical Journal	1	0.3	N/A
103	Npj Biofilms And Microbiomes	1	0.3	9.208
104	Virulence	1	0.3	N/A
105	Heliyon	1	0.3	N/A
106	Critical Reviews In Oncology Hematology	1	0.3	6.058
107	Antimicrobial Agents And Chemotherapy	1	0.3	4.2
108	Pharmaceuticals	1	0.3	4.573
109	Critical Reviews In Microbiology	1	0.3	N/A
110	Bioactive Materials	1	0.3	N/A
111	Bmc Veterinary Research	1	0.3	2.610
112	Applied Sciences-Basel	1	0.3	N/A
113	Journal Of Biomedical Materials Research Part A	1	0.3	4.606
114	Food Science And Human Wellness	1	0.3	N/A

Rank	Journal	Publications	Percentage (%)	Impact Factor
115	Biological Control	1	0.3	4.186
116	Journal Of Food Science	1	0.3	3.805
117	Journal Of Virology	1	0.3	5.277
118	Biotechnology Advances	1	0.3	15.704
119	Analytical Chemistry	1	0.3	7.421
120	Clinical Microbiology And Infection	1	0.3	13.793
121	Journal Of Microbiological Methods	1	0.3	2.125

Table S4. Journals of publications affiliated with the United States (133 journals). Impact factors from the 2025 JCR release (released June 2026).

Rank	Journal	Publications	Percentage (%)	Impact Factor
1	Viruses	21	6.5	3.800
2	Frontiers In Microbiology	15	4.7	5.800
3	Antimicrobial Agents And Chemotherapy	15	4.7	4.2
4	Applied And Environmental Microbiology	13	4.0	4.200
5	Antibiotics	12	3.7	5.5
6	Nature Communications	11	3.4	17.694
7	Plos One	10	3.1	2.800
8	Microbiology Spectrum	10	3.1	4.100
9	Scientific Reports	9	2.8	4.900
10	Journal Of Food Protection	9	2.8	1.984
11	Microorganisms	8	2.5	4.700
12	Foodborne Pathogens And Disease	7	2.2	2.369

Rank	Journal	Publications	Percentage (%)	Impact Factor
13	Poultry Science	6	1.9	4.357
14	Journal Of Dairy Science	5	1.6	3.448
15	Food Microbiology	5	1.6	5.312
16	Journal Of Food Safety	5	1.6	2.193
17	Mbio	4	1.2	6.265
18	Food Research International	4	1.2	8.039
19	Food Control	4	1.2	7.000
20	Lwt-Food Science And Technology	3	0.9	5.882
21	Nature Medicine	3	0.9	52.5
22	Frontiers In Cellular And Infection Microbiology	3	0.9	5.599
23	Msphere	3	0.9	4.729
24	Meat Science	3	0.9	6.958
25	Microbial Biotechnology	3	0.9	5.468
26	International Journal Of Molecular Sciences	3	0.9	6.208
27	Pathogens	3	0.9	3.640
28	Journal Of Molecular Biology	2	0.6	5.545
29	Current Opinion In Microbiology	2	0.6	5.426
30	Gut Microbes	2	0.6	12.220
31	Plos Computational Biology	2	0.6	4.169
32	Future Microbiology	2	0.6	2.5
33	Environmental Microbiology	2	0.6	4.909
34	Journal Of Crohns & Colitis	2	0.6	7.773

Rank	Journal	Publications	Percentage (%)	Impact Factor
35	Journal Of Environmental Quality	2	0.6	2.403
36	Canadian Journal Of Microbiology	2	0.6	2.750
37	Journal Of Bacteriology	2	0.6	3.099
38	Msystems	2	0.6	6.207
39	Annual Review Of Medicine	2	0.6	10.316
40	Avian Diseases	2	0.6	1.343
41	Microbiological Research	2	0.6	6.674
42	Journal Of Animal Science	2	0.6	3.076
43	Foods	2	0.6	5.160
44	Journal Of Animal Science And Biotechnology	2	0.6	6.927
45	Pharmaceuticals	2	0.6	4.573
46	International Journal Of Food Microbiology	2	0.6	5.700
47	Injury-International Journal Of The Care Of The Injured	1	0.3	2.388
48	Biological Control	1	0.3	4.186
49	Applied Microbiology And Biotechnology	1	0.3	4.931
50	Journal Of Clinical Investigation	1	0.3	15.669
51	Molecular Biology And Evolution	1	0.3	10.616
52	Surgical Infections	1	0.3	1.915
53	Pediatric Pulmonology	1	0.3	3.107
54	Life Sciences	1	0.3	6.024
55	Cell Systems	1	0.3	9.109
56	European Journal Of Immunology	1	0.3	5.585

Rank	Journal	Publications	Percentage (%)	Impact Factor
57	World Rabbit Science	1	0.3	0.843
58	Journal Of Food Science	1	0.3	3.805
59	Cellulose	1	0.3	5.666
60	Evolutionary Applications	1	0.3	3.951
61	Phytomedicine	1	0.3	7.729
62	Respiration	1	0.3	3.604
63	Molecular Medicine	1	0.3	5.652
64	Nature Microbiology	1	0.3	28.067
65	Accounts Of Chemical Research	1	0.3	18.200
66	Lancet Microbe	1	0.3	36.916
67	Journal Of Cosmetic Science	1	0.3	0.383
68	Animals	1	0.3	2.984
69	Bmc Genomics	1	0.3	4.312
70	Archives Of Pathology & Laboratory Medicine	1	0.3	4.551
71	Plos Neglected Tropical Diseases	1	0.3	3.624
72	Journal Of Surgical Research	1	0.3	2.135
73	Bioengineered	1	0.3	4.816
74	Journal Of The Pediatric Infectious Diseases Society	1	0.3	3.199
75	Journal Of Aquatic Animal Health	1	0.3	1.234
76	Mabs	1	0.3	5.172
77	Analytical Chemistry	1	0.3	7.421
78	Current Hiv/Aids Reports	1	0.3	4.591

Rank	Journal	Publications	Percentage (%)	Impact Factor
79	Letters In Applied Microbiology	1	0.3	2.309
80	Pediatric Infectious Disease Journal	1	0.3	3.512
81	Virology Journal	1	0.3	4.752
82	Molecular Microbiology	1	0.3	3.561
83	Science Advances	1	0.3	13.409
84	Food And Bioprocess Technology	1	0.3	5.592
85	Microbiologyopen	1	0.3	3.333
86	Infection And Immunity	1	0.3	3.037
87	Advances In Virus Research, Vol 111	1	0.3	3.436
88	Nutrients	1	0.3	5.804
89	Cell Biochemistry And Function	1	0.3	3.627
90	Cancer Immunology Immunotherapy	1	0.3	5.670
91	Porcine Health Management	1	0.3	3.382
92	Cell Host & Microbe	1	0.3	23.2
93	Biology-Basel	1	0.3	4.120
94	Microbiology-Sgm	1	0.3	2.594
95	Journal Of Industrial Microbiology & Biotechnology	1	0.3	3.304
96	Journal Of Clinical Microbiology	1	0.3	9.169
97	Virus Genes	1	0.3	1.667
98	Clinical Microbiology And Infection	1	0.3	13.793
99	Microbiology And Immunology	1	0.3	2.292
100	Hortscience	1	0.3	1.863

Rank	Journal	Publications	Percentage (%)	Impact Factor
101	Infectious Diseases	1	0.3	5.442
102	Microbial Genomics	1	0.3	3.862
103	Bmc Microbiology	1	0.3	4.213
104	Current Pharmaceutical Biotechnology	1	0.3	2.638
105	Bulletin Of Mathematical Biology	1	0.3	3.416
106	Advances In Applied Microbiology, Vol 78	1	0.3	4.500
107	Peerj	1	0.3	2.679
108	International Journal Of Pharmaceutics	1	0.3	5.759
109	Water Research	1	0.3	12.762
110	European Journal Of Clinical Microbiology & Infectious Diseases	1	0.3	4.567
111	Journal Of Invertebrate Pathology	1	0.3	3.457
112	Annual Review Of Microbiology, Vol 73	1	0.3	10.458
113	Environmental Science-Processes & Impacts	1	0.3	5.501
114	Iscience	1	0.3	5.740
115	Disease Models & Mechanisms	1	0.3	4.105
116	Journal Of Applied Microbiology	1	0.3	3.582
117	Molecular Biology Reports	1	0.3	2.823
118	Current Microbiology	1	0.3	2.622
119	Irish Journal Of Agricultural And Food Research	1	0.3	N/A
120	Annual Review Of Virology, Vol 7, 2020	1	0.3	11.170
121	Frontiers In Veterinary Science	1	0.3	3.181
122	Aquaculture	1	0.3	4.444

Rank	Journal	Publications	Percentage (%)	Impact Factor
123	Science	1	0.3	55.773
124	Microbial Pathogenesis	1	0.3	3.764
125	Virus Research	1	0.3	4.950
126	Journal Of Virology	1	0.3	5.277
127	Environmental Science & Technology Letters	1	0.3	10.850
128	Medicina-Lithuania	1	0.3	2.513
129	Journal Of Medical Microbiology	1	0.3	2.822
130	Journal Of Microbiological Methods	1	0.3	2.125
131	Virologica Sinica	1	0.3	5.542
132	Livestock Science	1	0.3	1.832
133	Nanotechnology	1	0.3	3.393

Table S5. Complete gene list for *Pseudomonas aeruginosa* PAO1: all 211 genes in the resistance (R), phage receptor/defense (P), and biofilm (B) gene sets, with per-gene membership (1, member; 0, non-member).

Gene	R	P	B
alg44	0	1	1
alg8	0	1	1
algA	0	0	1
algB	0	0	1
algC	0	0	1
algD	0	1	1
algE	0	0	1
algF	0	0	1

Gene	R	P	B
algG	0	0	1
algK	0	0	1
algL	0	0	1
algU	0	1	1
algX	0	0	1
ampC	1	0	0
ampD	1	0	0
ampG	1	0	0
ampR	1	0	0
aphA	1	0	0
arnA	1	1	0
arnB	1	1	0
arnC	1	1	0
arnD	1	1	0
arnE	1	1	0
arnF	1	1	0
arnG	1	0	0
arnT	1	1	0
bifA	0	0	1
cas1	0	1	0
cas2	0	1	0
cas3	0	1	0

Gene	R	P	B
csm1	0	1	0
csm2	0	1	0
csm3	0	1	0
csm4	0	1	0
csm5	0	1	0
csy1	0	1	0
csy2	0	1	0
csy3	0	1	0
csy4	0	1	0
cupA	0	0	1
cupB	0	0	1
cupC	0	0	1
dacB	1	0	0
dinB	1	1	0
fimV	0	1	0
flgE	0	1	1
fliC	0	1	1
fptA	0	1	0
fpvA	0	1	0
fpvB	0	1	0
gacA	0	0	1
gacS	0	0	1

Gene	R	P	B
galU	0	1	0
gyrA	1	0	0
gyrB	1	0	0
hsdM	0	1	0
hsdR	0	1	0
hsdS	0	1	0
lasI	0	0	1
lasR	0	0	1
lexA	1	1	0
lpxC	1	0	0
mexA	1	0	0
mexB	1	0	0
mexC	1	0	0
mexD	1	0	0
mexE	1	0	0
mexF	1	0	0
mexG	1	0	0
mexH	1	0	0
mexI	1	0	0
mexJ	1	0	0
mexK	1	0	0
mexL	1	0	0

Gene	R	P	B
mexM	1	0	0
mexN	1	0	0
mexP	1	0	0
mexQ	1	0	0
mexR	1	0	0
mexT	1	0	0
mexV	1	0	0
mexW	1	0	0
mexX	1	0	0
mexY	1	0	0
mexZ	1	0	0
motA	0	1	0
mucA	0	1	1
mucB	0	1	1
mucC	0	1	1
mucD	0	1	1
mutL	1	0	0
mutS	1	0	0
mutT	1	0	0
nalC	1	0	0
nalD	1	0	0
opmB	1	0	0

Gene	R	P	B
opmD	1	0	0
opmE	1	0	0
oprD	1	1	0
oprF	1	1	0
oprH	1	0	0
oprJ	1	0	0
oprM	1	0	0
oprN	1	0	0
pagP	1	0	0
parC	1	0	0
parE	1	0	0
pelA	0	1	1
pelB	0	1	1
pelC	0	1	1
pelD	0	1	1
pelE	0	1	1
pelF	0	1	1
pelG	0	1	1
pfeA	0	1	0
phoP	1	1	0
phoQ	1	1	0

Gene	R	P	B
pilA	0	1	1
pilB	0	1	0
pilC	0	1	0
pilD	0	1	0
pilE	0	1	0
pilM	0	1	0
pilQ	0	1	0
pilT	0	1	0
pilY1	0	0	1
piuA	0	1	0
pmrA	1	0	0
pmrB	1	1	0
pmrC	1	0	0
pmrD	1	0	0
pmrE	1	0	0
pmrF	1	0	0
pmrG	1	0	0
pmrH	1	0	0
pmrI	1	0	0
pmrJ	1	0	0
pmrK	1	0	0
pmrL	1	0	0

Gene	R	P	B
pmrM	1	0	0
pmrN	1	0	0
pqsA	0	0	1
pqsB	0	0	1
pqsC	0	0	1
pqsD	0	0	1
pqsE	0	0	1
pslA	0	1	1
pslB	0	0	1
pslC	0	0	1
pslD	0	1	1
pslE	0	1	1
pslF	0	1	1
pslG	0	0	1
pslH	0	0	1
pslI	0	1	1
pslJ	0	1	1
pslK	0	1	1
pslL	0	1	1
pslM	0	1	1
pslN	0	1	1
pslO	0	1	1

Gene	R	P	B
qscR	0	0	1
rbdA	0	0	1
recA	1	1	0
recN	1	0	0
rhlI	0	0	1
rhlR	0	0	1
rmlA	0	1	0
rmlB	0	1	0
rmlC	0	1	0
rmlD	0	1	0
roeA	0	0	1
rpoS	1	0	1
rsmA	0	0	1
rsmY	0	0	1
rsmZ	0	0	1
sadC	0	0	1
sulA	1	1	0
umuC	1	1	0
umuD	1	1	0
waaA	0	1	0
waaB	0	1	0
waaC	0	1	0

Gene	R	P	B
waaD	0	1	0
waaE	0	1	0
waaF	0	1	0
waaG	0	1	0
waaH	0	1	0
waaL	0	1	0
waaP	0	1	0
waaQ	0	1	0
waaR	0	1	0
wbpB	0	1	0
wbpE	0	1	0
wbpG	0	1	0
wbpL	0	1	0
wbpM	0	1	0
wbpW	0	1	0
wbpX	0	1	0
wbpY	0	1	0
wbpZ	0	1	0
wspA	0	0	1
wspB	0	0	1
wspC	0	0	1
wspD	0	0	1

Gene	R	P	B
wspE	0	0	1
wspF	0	0	1
wspR	0	0	1
wzy	0	1	0
wzz1	0	1	0
wzz2	0	1	0

Table S6. Complete gene list for *Escherichia coli* K-12 MG1655: all 184 genes in the resistance (R), phage receptor/defense (P), and biofilm (B) gene sets, with per-gene membership (1, member; 0, non-member).

Gene	R	P	B
acrA	1	0	0
acrB	1	0	0
acrD	1	0	0
acrE	1	0	0
acrF	1	0	0
acrS	1	0	0
acrZ	1	0	0
ampC	1	0	0
ampD	1	0	0
ampE	1	0	0
ampG	1	0	0
arnA	1	1	0
arnB	1	1	0

Gene	R	P	B
arnC	1	1	0
arnD	1	1	0
arnE	1	1	0
arnF	1	1	0
arnT	1	1	0
bcr	1	0	0
bcsA	0	0	1
bcsB	0	0	1
bcsC	0	0	1
bcsD	0	0	1
bcsE	0	0	1
bcsF	0	0	1
bcsG	0	0	1
bcsQ	0	0	1
bcsR	0	0	1
bcsS	0	0	1
bcsT	0	0	1
bcsU	0	0	1
bcsZ	0	0	1
bssR	0	0	1
bssS	0	0	1
btuB	0	1	0

Gene	R	P	B
cirA	0	1	0
cpxA	1	0	0
cpxR	1	0	0
csgA	0	0	1
csgB	0	0	1
csgC	0	0	1
csgD	0	0	1
csgE	0	0	1
csgF	0	0	1
csgG	0	0	1
dinB	1	1	0
dosC	0	0	1
dosD	0	0	1
emrA	1	0	0
emrB	1	0	0
emrD	1	0	0
emrE	1	0	0
eptA	1	1	0
eptB	1	1	0
fadL	0	1	0
fepA	0	1	0
fhuA	0	1	0

Gene	R	P	B
fimA	0	1	0
fimC	0	1	0
fimD	0	1	0
fimF	0	1	0
fimG	0	1	0
fimH	0	1	0
fimI	0	1	0
flgE	0	1	1
flgL	0	1	0
flhC	0	1	1
flhD	0	1	1
fliA	0	1	1
fliC	0	1	1
fliF	0	1	0
fliM	0	1	0
fliN	0	1	0
flu	0	1	0
gyrA	1	0	0
gyrB	1	0	0
hsdM	0	1	0
hsdR	0	1	0
hsdS	0	1	0

Gene	R	P	B
lamB	0	1	0
lexA	1	1	0
lit	0	1	0
lpxA	0	1	0
lpxB	0	1	0
lpxC	0	1	0
lpxD	0	1	0
lpxH	0	1	0
lpxK	0	1	0
lpxL	0	1	0
lpxM	0	1	0
lpxO	1	1	0
lpxP	0	1	0
marA	1	0	0
marB	1	0	0
marR	1	0	0
mcrA	0	1	0
mcrB	0	1	0
mcrC	0	1	0
mdfA	1	0	0
mdtA	1	0	0
mdtB	1	0	0

Gene	R	P	B
mdtC	1	0	0
mdtG	1	0	0
mdtH	1	0	0
mdtK	1	0	0
mdtL	1	0	0
mdtM	1	0	0
mgrB	1	1	0
mlrA	0	0	1
motA	0	1	1
motB	0	1	1
mutL	1	0	0
mutS	1	0	0
mutT	1	0	0
nmpC	0	1	0
ompA	0	1	0
ompC	1	1	0
ompF	1	1	0
ompT	0	1	0
ompW	0	1	0
ompX	0	1	0
pagP	1	1	0
parC	1	0	0

Gene	R	P	B
parE	1	0	0
pdeH	0	0	1
pgaA	0	0	1
pgaB	0	0	1
pgaC	0	0	1
pgaD	0	0	1
phoP	1	1	0
phoQ	1	1	0
pifA	0	1	0
pmrA	1	1	0
pmrB	1	1	0
pmrD	1	0	0
recA	1	1	0
recF	1	1	0
recN	1	1	0
rob	1	0	0
rpoS	0	0	1
soxR	1	0	0
soxS	1	0	0
sulA	1	1	0
tolC	1	0	0
traA	0	1	0

Gene	R	P	B
tsx	0	1	0
umuC	1	1	0
umuD	1	1	0
waaA	0	1	0
waaB	0	1	0
waaC	0	1	0
waaD	0	1	0
waaE	0	1	0
waaF	0	1	0
waaG	0	1	0
waaH	0	1	0
waaL	0	1	0
waaP	0	1	0
waaQ	0	1	0
waaR	0	1	0
waaU	0	1	0
waaV	0	1	0
waaW	0	1	0
waaY	0	1	0
waaZ	0	1	0
wcaA	0	1	0
wcaB	0	1	0

Gene	R	P	B
wcaC	0	1	0
wcaD	0	1	0
wcaE	0	1	0
wcaF	0	1	0
wcaG	0	1	0
wcaH	0	1	0
wcaI	0	1	0
wcaJ	0	1	0
wcaK	0	1	0
wzx	0	1	0
wzy	0	1	0
wzzE	0	1	0
yciR	0	0	1
yhiUV	1	0	0
yhjH	0	0	1
ymgA	0	0	1
ymgB	0	0	1

Table S7. Annual publication output and citations of the 1,598-record corpus (1992–2026).

Year	Publications	Cumulative publications	Total citations	Citations per paper
1992	1	1	18	18.0
1995	1	2	12	12.0
1997	1	3	38	38.0

1998	2	5	51	25.5
2002	1	6	32	32.0
2003	1	7	194	194.0
2004	4	11	852	213.0
2005	4	15	412	103.0
2006	6	21	386	64.3
2007	8	29	640	80.0
2008	11	40	727	66.1
2009	12	52	959	79.9
2010	18	70	1821	101.2
2011	26	96	2277	87.6
2012	17	113	1376	80.9
2013	26	139	2126	81.8
2014	40	179	1936	48.4
2015	33	212	2063	62.5
2016	50	262	3063	61.3
2017	64	326	5473	85.5
2018	65	391	4131	63.6
2019	83	474	7015	84.5
2020	99	573	4234	42.8
2021	152	725	5417	35.6
2022	175	900	4882	27.9
2023	153	1053	3095	20.2
2024	181	1234	2905	16.0
2025	213	1447	1082	5.1
2026	151	1598	137	0.9
Total (1992–2026)	1598			

Times cited are the Web of Science "Times-Cited" values in the export of 5 July 2026. One record in the export carries no Times-Cited value and is therefore not represented in the citation columns; publication counts are complete. Citation totals change as the database is updated.

Table S8. KEGG Orthology (KO) verification of the 48 PAO1 candidate genes. Gene symbols are italic. "Corroborated" indicates that at least one KO identifier is shared with a K-12 candidate gene.

PAO1 candidate gene	KEGG Orthology (PAO1)	Corroborated by shared KO	Shared KO identifier(s)

alg44	ko:K19291	no	NA
alg8	ko:K19290	no	NA
algd	ko:K00066	no	NA
algu	ko:K03088	no	NA
arna	ko:K10011	yes	ko:K10011
arnb	ko:K07806	yes	ko:K07806
arnc	ko:K10012	yes	ko:K10012
arnd	ko:K13014	yes	ko:K13014
arne	ko:K12962	yes	ko:K12962
arnf	ko:K12963	yes	ko:K12963
arnt	ko:K07264	yes	ko:K07264
dinb	ko:K02346	yes	ko:K02346
flge	ko:K02390	yes	ko:K02390
flic	ko:K02406	yes	ko:K02406
lexa	ko:K01356	yes	ko:K01356
muca	ko:K03597	no	NA
mucb	ko:K03598	no	NA
mucc	ko:K03803	no	NA
mucd	ko:K04771	no	NA
oprd	ko:K18093	no	NA
oprf	ko:K03286	no	NA
pela	ko:K21006	no	NA
pelb	ko:K21007	no	NA
pelc	ko:K21008	no	NA
peld	ko:K21009	no	NA
pele	ko:K21010	no	NA
pelf	ko:K21011	no	NA
pelg	ko:K21012	no	NA
phop	ko:K07660	yes	ko:K07660
phoq	ko:K07637	yes	ko:K07637
pila	ko:K02650	no	NA
pmrb	ko:K07645	no	NA
psla	ko:K20997	no	NA

psld	ko:K20987	no	NA
psle	ko:K20998	no	NA
pslf	ko:K20999	no	NA
psli	ko:K21002	no	NA
pslj	ko:K21003	no	NA
pslk	ko:K21004	no	NA
psll	ko:K21005	no	NA
pslm	NA	no	NA
psln	ko:K03168	no	NA
pslo	NA	no	NA
reca	ko:K03553	yes	ko:K03553
rpos	ko:K03087	no	NA
sula	NA	no	NA
umuc	NA	no	NA
umud	NA	no	NA

KO assignments were retrieved through the KEGG REST API. Four of the 18 symbol-level matches could not be corroborated: pmrB (no KO for the K-12 entry) and sulA, umuC and umuD (no KO assignment in PAO1).

Table S9. Specificity control. Overlap of the resistance (R), phage receptor/defense (P), and biofilm (B) gene sets with four unrelated control gene sets of comparable size, tested by the same one-sided hypergeometric test against the same species background. Odds ratios (OR) and uncorrected P values are shown; n is the intersection size.

**Species**	**Control gene set**	**R ∩ control**	**P ∩ control**	**B ∩ control**
PAO1	central metabolism (glycolysis+TCA+oxidative phosphorylation)	n = 0; OR = 0.00; P = 1.000	n = 0; OR = 0.00; P = 1.000	n = 1; OR = 0.68; P = 0.772
PAO1	ribosome / translation machinery	n = 0; OR = 0.00; P = 1.000	n = 0; OR = 0.00; P = 1.000	n = 0; OR = 0.00; P = 1.000
PAO1	bacterial secretion system (envelope-proximal)	n = 0; OR = 0.00; P = 1.000	n = 0; OR = 0.00; P = 1.000	n = 0; OR = 0.00; P = 1.000
PAO1	purine metabolism	n = 0; OR = 0.00; P = 1.000	n = 0; OR = 0.00; P = 1.000	n = 1; OR = 1.18; P = 0.578
K-12	central metabolism (glycolysis+TCA+oxidative phosphorylation)	n = 0; OR = 0.00; P = 1.000	n = 0; OR = 0.00; P = 1.000	n = 0; OR = 0.00; P = 1.000
K-12	ribosome / translation	n = 0; OR = 0.00; P	n = 0; OR = 0.00; P	n = 0; OR = 0.00; P

	machinery	= 1.000	= 1.000	= 1.000
K-12	bacterial secretion system (envelope-proximal)	n = 1; OR = 1.92; P = 0.417	n = 0; OR = 0.00; P = 1.000	n = 0; OR = 0.00; P = 1.000
K-12	purine metabolism	n = 0; OR = 0.00; P = 1.000	n = 0; OR = 0.00; P = 1.000	n = 0; OR = 0.00; P = 1.000
**Species**	**Overlap**	**Intersection (n)**	**OR**	**P (one-sided hypergeometric)**
PAO1	R ∩ P	18	17.74	3.50e-15
PAO1	P ∩ B	29	47.02	1.52e-32
K-12	R ∩ P	26	30.16	4.88e-25
K-12	P ∩ B	7	8.08	7.63e-05

Observed overlaps (second block) are reproduced here for direct comparison. Control gene sets were retrieved from KEGG: central metabolism (glycolysis, TCA cycle, oxidative phosphorylation), ribosome/translation, bacterial secretion system, and purine metabolism.

Table S10. Sensitivity of the overlap enrichment to pre-existing dual annotation. Genes already described as dual-function in the prior literature (LPS-modification and exopolysaccharide-matrix modules) were removed from all three gene sets before the overlap tests were repeated (36 genes in PAO1, 16 in K-12).

Species	Analysis	Overlap	|A|	|B|	Intersection	OR	P	Genes removed
PAO1	Full analysis (as reported)	R ∩ P	81	105	18	17.74	3.50e-15	36
PAO1	Full analysis (as reported)	P ∩ B	105	73	29	47.02	1.52e-32	36
PAO1	Sensitivity: dual-annotated module removed	R ∩ P	71	69	8	11.32	2.09e-06	36
PAO1	Sensitivity: dual-annotated module removed	P ∩ B	69	47	3	5.64	2.00e-02	36
K-12	Full analysis (as reported)	R ∩ P	69	106	26	30.16	4.88e-25	16

K-12	Full analysis (as reported)	P ∩ B	106	42	7	8.08	7.63e-05	16
K-12	Sensitivity: dual-annotated module removed	R ∩ P	53	90	10	11.65	1.29e-07	16
K-12	Sensitivity: dual-annotated module removed	P ∩ B	90	42	7	9.67	2.64e-05	16

Composition of the observed overlaps (all genes at least partially dual-annotated in the prior literature).

**Species**	**Overlap**	**Component 1**	**Component 2**	**Component 3**
PAO1	R ∩ P (n = 18)	LPS-modification / two-component: 10 (55.6%)	SOS / error-prone repair: 6 (33.3%)	Porin / outer membrane: 2 (11.1%)
PAO1	P ∩ B (n = 29)	EPS matrix polysaccharide (alginate/Pel/Psl): 22 (75.9%)	Alginate regulators (mucABCD): 4 (13.8%)	Surface appendages (flagella/pili): 3 (10.3%)
K-12	R ∩ P (n = 26)	LPS-modification / two-component: 16 (61.5%)	SOS / error-prone repair: 8 (30.8%)	Porin / outer membrane: 2 (7.7%)

The P ∩ B overlap in PAO1 is therefore largely definitional, whereas the R ∩ P overlap persists after removal of dual-annotated genes in both species.

Table S11. Complete gene list for Acinetobacter baumannii ATCC 17978: all 317 genes in the resistance (R), phage receptor/defense (P), and biofilm (B) sets, with per-gene membership (1 = member, 0 = non-member). Locus tags follow the ATCC 17978 annotation (taxonomy ID 400667); gene symbols and product descriptions were taken from KEGG (organism code acb) and from RefSeq product names, because this strain has no widely used gene-symbol namespace. Set sizes: R, n = 96; P, n = 213; B, n = 45.

Locus tag	Gene symbol / product	R	P	B
A1S_0003	DNA replication recombination and repair protein	0	1	0
A1S_0004	DNA gyrase	1	0	0
A1S_0008	putative RND type efflux pump	1	0	0
A1S_0009	putative RND type efflux	1	0	0

	pump			
A1S_0010	RND type efflux pump	1	0	0
A1S_0016	Site-specific tyrosine recombinase	1	0	0
A1S_0049	Protein tyrosine kinase	0	1	0
A1S_0052	WecC protein	0	1	0
A1S_0053	MviM protein	0	1	0
A1S_0054	WbbJ protein	0	1	0
A1S_0055	WecE protein	0	1	0
A1S_0056	O-antigen translocase	0	1	0
A1S_0057	Capsular polysaccharide synthesis enzyme	0	0	1
A1S_0059	putative glycosyltransferase	0	1	0
A1S_0062	putative UTP-glucose-1-phosphate uridylyltransferase	0	1	0
A1S_0066	hypothetical protein	0	1	0
A1S_0092	putative ferric siderophore receptor protein	0	1	0
A1S_0097	hypothetical protein	0	0	1
A1S_0142	Homoserine/homoserine lactone efflux protein	1	0	0
A1S_0185	hypothetical protein	0	0	1
A1S_0186	DNA polymerase IV	0	1	0
A1S_0201	putative outer membrane protein	0	1	0
A1S_0228	Putative membrane protein	0	1	0
A1S_0229	Putative membrane protein	0	1	0
A1S_0232	type 4 fimbriae expression regulatory protein	0	1	0

A1S_0233	type 4 fimbriae expression regulatory protein	0	1	0
A1S_0234	type 4 fimbriae expression regulatory protein	0	1	0
A1S_0241	Site-specific tyrosine recombinase	1	0	0
A1S_0255	Putative RND family drug transporter	1	1	0
A1S_0261	Alginate biosynthesis regulatory protein	0	1	1
A1S_0266	Putative membrane protein	0	1	0
A1S_0310	EsvL	0	1	0
A1S_0315	DNA repair system	1	0	0
A1S_0321	Recombination and DNA repair protein	1	1	0
A1S_0327	type 4 prepilin-like proteins leader peptide processing enzyme	0	1	0
A1S_0328	Type 4 fimbrial assembly protein	0	1	1
A1S_0329	type 4 fimbrial biogenesis protein	0	1	1
A1S_0339	pH adaptation potassium efflux system protein G	1	0	0
A1S_0359	putative beta-lactamase	1	0	0
A1S_0365	putative amino-acid efflux transmembrane protein	1	0	0
A1S_0375	Magnesium and cobalt efflux protein	1	0	0
A1S_0395	Na+-driven multidrug efflux pump	1	0	0
A1S_0396	Putative membrane protein	0	1	0
A1S_0430	putative	0	1	0

	glycosyltransferase			
A1S_0431	lipid A biosynthesis lauroyl acyltransferase	1	1	0
A1S_0474	putative ferric siderophore receptor protein	0	1	0
A1S_0494	putative glycosyl transferase	0	1	0
A1S_0495	putative glycosyl transferase	0	1	0
A1S_0500	type 4 fimbrial biogenesis protein	0	1	1
A1S_0504	hypothetical protein	0	0	1
A1S_0535	Putative RND family drug transporter	1	0	0
A1S_0536	macrolide transport protein	1	0	0
A1S_0537	Putative RND family drug transporter	1	0	0
A1S_0538	Putative RND family drug transporter	1	0	0
A1S_0551	hypothetical protein	0	1	0
A1S_0595	Putative membrane protein	0	1	0
A1S_0607	exopolyphosphatase	0	1	1
A1S_0635	hypothetical bacteriophage protein	0	1	0
A1S_0636	DNA polymerase V component	0	1	0
A1S_0637	DNA-directed DNA polymerase	0	1	0
A1S_0643	hypothetical protein	0	1	0
A1S_0649	Putative phage primase	0	1	0
A1S_0655	putative efflux protein	1	0	0
A1S_0661	Phage integrase family	0	1	0

	protein			
A1S_0662	Phage integrase family protein	0	1	0
A1S_0693	FilD	0	1	0
A1S_0709	Putative cation efflux system protein	1	0	0
A1S_0710	putative SMR family drug transporter	1	0	0
A1S_0730	putative long-chain fatty acid transport protein	0	1	0
A1S_0740	putative phage related protein	0	1	0
A1S_0751	Diguanylate cyclase	0	0	1
A1S_0777	putative threonine efflux protein (RhtC)	1	0	0
A1S_0847	putative signal peptide	0	1	0
A1S_0860	hypothetical protein	0	1	0
A1S_0877	threonine efflux protein	1	0	0
A1S_0884	putative outer membrane protein	0	1	0
A1S_0887	phosphomannomutase	0	1	0
A1S_0896	Twitching motility protein	0	1	0
A1S_0897	Twitching motility protein	0	1	0
A1S_0932	Putative membrane protein	0	1	1
A1S_0939	Putative transport protein	0	0	1
A1S_0959	putative signal peptide	0	1	0
A1S_0980	Ferric enterobactin receptor precursor	0	1	0
A1S_0981	Ferric enterobactin receptor precursor	0	1	0
A1S_1008	isocitrate lyase	0	0	1
A1S_1020	Penicillin-binding protein 2	1	0	0

A1S_1030	DNA-binding ATP-dependent protease La	0	1	1
A1S_1031	DNA-binding ATP-dependent protease La	0	1	1
A1S_1033	Putative antigen	0	1	0
A1S_1044	Co/Zn/Cd efflux system	1	0	0
A1S_1045	Co/Zn/Cd efflux system	1	0	0
A1S_1063	TonB-dependent siderophore receptor	0	1	0
A1S_1074	hypothetical protein	0	0	1
A1S_1107	Porin	0	1	0
A1S_1118	putative porin for vanillate trafficking (VanP)	0	1	0
A1S_1144	merops peptidase family S24	0	1	0
A1S_1146	site-specific DNA-methyltransferase	0	1	0
A1S_1153	putative phage-related protein	0	1	0
A1S_1154	Putative bacteriophage protein	0	1	0
A1S_1155	putative phage-related protein	0	1	0
A1S_1164	Putative phage tail tape meausure protein	0	1	0
A1S_1165	Putative phage tail tape meausure protein	0	1	0
A1S_1173	RumB	0	1	0
A1S_1174	DNA polymerase V component	0	1	0
A1S_1175	phage integrase	0	1	0
A1S_1193	OmpA/MotB	0	1	0
A1S_1220	Putative threonine efflux protein	1	0	0

A1S_1230	phage putative head morphogenesis protein	0	1	0
A1S_1236	D-arabinose 5-phosphate isomerase	0	1	0
A1S_1237	3-Deoxy-D-manno-octulosonate 8-phosphate (KDO 8-P) phosphatase	0	1	0
A1S_1238	hypothetical protein	0	1	0
A1S_1239	Putative transport protein	0	1	0
A1S_1241	Putative RND family drug transporter	1	0	0
A1S_1253	putative permease	1	0	0
A1S_1254	hypothetical protein	0	0	1
A1S_1255	lipid A biosynthesis lauroyl acyltransferase	1	1	0
A1S_1264	putative class A beta-lactamase	1	0	0
A1S_1290	hypothetical protein	0	1	0
A1S_1305	Putative outer membrane lipoprotein	0	1	0
A1S_1306	hypothetical protein	0	1	0
A1S_1380	Putative protein (DcaP-like)	0	1	0
A1S_1385	hypothetical protein	0	1	1
A1S_1389	DNA polymerase V component	0	1	0
A1S_1402	putative amino acid efflux transmembrane protein	1	0	0
A1S_1441	putative signal peptide	0	1	0
A1S_1494	Putative protein (DcaP-like)	0	1	0
A1S_1501	Phage integrase	0	1	0
A1S_1503	transmembrane pair	1	0	0
A1S_1507	Fimbrial protein	0	1	1

A1S_1508	Fimbrial biogenesis outer membrane usher protein	0	1	1
A1S_1509	pili assembly chaperone	0	1	1
A1S_1510	Fimbrial protein	0	1	1
A1S_1517	Beta-lactamase OXA-95	1	0	0
A1S_1518	putative suppressor of F exclusion of phage T7	0	1	0
A1S_1546	Organic solvent tolerance protein precursor	0	1	0
A1S_1547	Organic solvent tolerance protein precursor	0	1	0
A1S_1556	tetraacyldisaccharide 4'-kinase	0	1	0
A1S_1557	3-deoxy-manno-octulosonate cytidylyltransferase	0	1	0
A1S_1559	type 4 fimbrial biogenesis protein	0	1	1
A1S_1574	Putative membrane protein	0	1	0
A1S_1576	Putative membrane protein	1	0	0
A1S_1582	Transcriptional regulator Cro/CI family	0	1	0
A1S_1588	Phage terminase-like protein large subunit	0	1	0
A1S_1590	peptidase U35 phage prohead HK97	0	1	0
A1S_1591	Phage major capsid protein HK97	0	1	0
A1S_1592	Putative Phage head-tail adaptor	0	1	0
A1S_1597	phage tail tape meausure protein lambda family	0	1	0
A1S_1600	Lysozyme	0	1	0

A1S_1607	TonB-dependent receptor	0	1	0
A1S_1655	putative ferric siderophore receptor protein	0	1	0
A1S_1667	putative ferric hydroxamate siderophore receptor	0	1	0
A1S_1668	Lipid A-disaccharide synthase	1	1	0
A1S_1677	putative porin precursor	0	1	0
A1S_1695	Putative two-component response regulator	0	0	1
A1S_1708	Beta-lactamase-like protein	1	0	0
A1S_1725	putative ferric siderophore receptor protein	0	1	0
A1S_1749	ABC transporter-like protein	0	1	0
A1S_1750	AdeB	1	0	0
A1S_1753	AdeR	1	0	0
A1S_1754	AdeS	1	0	0
A1S_1755	AdeT	1	0	0
A1S_1760	hypothetical protein	0	1	0
A1S_1769	Putative RND family drug transporter	1	0	0
A1S_1799	Multidrug efflux MFS transporter putative	1	0	0
A1S_1802	hypothetical protein	0	1	0
A1S_1803	hypothetical protein	0	1	0
A1S_1816	putative long-chain fatty acid transport protein	0	1	0
A1S_1853	hypothetical protein	0	1	0
A1S_1881	Porin	0	1	0
A1S_1899	2-dehydro-3-deoxyphosphooctonate	0	1	0

	aldolase			
A1S_1962	DNA strand exchange and recombination protein	0	1	0
A1S_1965	UDP-acetylglucosamine acyltransferase	0	1	0
A1S_1966	hypothetical protein	0	1	0
A1S_1967	hypothetical protein	0	1	0
A1S_2008	DNA repair protein	1	1	0
A1S_2015	DNA-directed DNA polymerase	0	1	0
A1S_2016	Phage-related lysozyme	0	1	0
A1S_2028	phage putative head morphogenesis protein	0	1	0
A1S_2034	hypothetical protein	0	1	0
A1S_2036	DNA cytosine methyltransferase	0	1	0
A1S_2037	EsvI	0	1	0
A1S_2040	Putative phage integrase	0	1	0
A1S_2063	Putative membrane protein	1	0	0
A1S_2080	putative siderophore receptor	0	1	0
A1S_2081	TonB-dependent siderophore receptor	0	1	0
A1S_2088	hypothetical protein	0	1	0
A1S_2089	Fimbrial usher protein	0	1	1
A1S_2091	Putative exported protein	0	1	0
A1S_2110	UDP-23-diacylglucosamine hydrolase	0	1	0
A1S_2160	haemin storage system HmsR protein	0	0	1
A1S_2198	putative mutlidrug resistance protein	1	0	0

A1S_2201	hypothetical protein	0	1	0
A1S_2213	CsuE	0	1	1
A1S_2214	CsuD	0	1	1
A1S_2215	CsuC	0	0	1
A1S_2216	CsuB	0	1	1
A1S_2217	CsuA	0	1	1
A1S_2218	CsuA/B	0	1	1
A1S_2224	threonine efflux protein	1	0	0
A1S_2298	SMR family efflux pump	1	0	0
A1S_2305	Cation/multidrug efflux pump	1	0	0
A1S_2306	Putative RND family drug transporter	1	0	0
A1S_2357	putative TonB-dependent receptor	0	1	0
A1S_2358	putative TonB-dependent receptor	0	1	0
A1S_2367	betalactamase	1	0	0
A1S_2372	Putative acinetobactin biosynthesis protein	0	1	0
A1S_2373	Putative acinetobactin biosynthesis protein	0	1	0
A1S_2374	Putative acinetobactin biosynthesis protein	0	1	0
A1S_2379	Putative acinetobactin biosynthesis protein	0	1	0
A1S_2380	Putative acinetobactin biosynthesis protein	0	1	0
A1S_2381	Putative acinetobactin biosynthesis protein	0	1	0
A1S_2383	Putative acinetobactin biosynthesis protein	0	1	0
A1S_2385	putative ferric acinetobactin receptor	0	1	0

A1S_2386	Putative ferric acinetobactin binding protein	0	1	0
A1S_2390	Putative acinetobactin biosynthesis protein	0	1	0
A1S_2391	Putative acinetobactin biosynthesis protein	0	1	0
A1S_2392	Putative acinetobactin utilization protein	0	1	0
A1S_2448	Putative phosphate transporter	0	0	1
A1S_2450	putative pyruvate decarboxylase	0	0	1
A1S_2461	Putative membrane protein	1	0	0
A1S_2489	UDP-N-acetyl glucosamine-2-epimerase	0	1	0
A1S_2504	excinuclease ABC subunit B	1	1	0
A1S_2506	Putative GGDEF family protein	0	0	1
A1S_2517	DNA repair protein	1	0	0
A1S_2521	Leader peptidase	1	0	0
A1S_2538	outer membrane protein CarO precursor	1	0	0
A1S_2562	Putative multidrug resistance pump	1	0	0
A1S_2566	putative ferric siderophore receptor protein	0	1	0
A1S_2587	holliday junction helicase subunit A	0	1	0
A1S_2588	holliday junction helicase subunit B	0	1	0
A1S_2595	peptidoglycan-associated lipoprotein precursor	0	1	0
A1S_2601	putative outer membrane	1	0	0

	protein A			
A1S_2604	Putative permease (PerM family)	1	0	0
A1S_2606	Phosphoribosylglycinamide formyltransferase 1	0	1	1
A1S_2609	putative lipid A biosynthesis lauroyl acyltransferase	0	1	0
A1S_2618	Putative RND family drug transporter	1	0	0
A1S_2619	Putative RND family drug transporter	1	0	0
A1S_2624	putative CPS-53 prophage bactoprenol glucosyl transferase	0	1	0
A1S_2625	Putative membrane protein	0	1	0
A1S_2626	DNA gyrase	1	0	0
A1S_2629	Site-specific tyrosine recombinase	1	0	0
A1S_2634	DNA repair protein	1	0	0
A1S_2660	Putative RND family drug transporter	1	0	0
A1S_2735	AdeI	1	0	0
A1S_2736	RND family drug transporter	1	0	0
A1S_2737	AdeK	1	0	0
A1S_2752	Lipid A phosphoethanolamine transferase	1	1	0
A1S_2753	Putative protein (DcaP-like)	0	1	0
A1S_2768	bacitracin resistance protein	1	0	0
A1S_2773	putative long-chain fatty	0	1	0

	acid transport protein			
A1S_2829	putative tonB-dependent receptor protein	0	1	0
A1S_2830	DNA-3-methyladenine glycosylase	1	0	0
A1S_2835	GTP-binding elongation factor family protein	0	1	1
A1S_2840	Outer membrane protein A	1	0	0
A1S_2841	putative type 4 fimbrial biogenesis protein FimT	0	1	1
A1S_2844	quaternary ammonium compound-resistance protein	1	0	0
A1S_2849	Putative glucose-sensitive porin (OprB-like)	0	1	0
A1S_2877	Vitamin B12 receptor precursor	0	1	0
A1S_2892	putative TonB-dependent receptor protein	0	1	0
A1S_2897	UDP-N-acetylglucosamine 2-epimerase	0	1	1
A1S_2899	putative glycosyltransferase	0	1	0
A1S_2900	putative lipopolysaccharide core biosynthesis glycosyl transferase LpsC	0	1	0
A1S_2918	DNA repair protein	1	0	0
A1S_2927	Phage integrase	0	1	0
A1S_2928	hypothetical protein	0	1	0
A1S_2929	Putative cation efflux system protein	1	0	0
A1S_2932	Heavy metal efflux pump CzcA	1	0	0
A1S_2934	heavy metal RND efflux	1	0	0

	outer membrane protein CzcC family			
A1S_2987	Putative lipoprotein precursor	0	1	0
A1S_2988	Putative membrane protein	0	1	0
A1S_3037	Putative ribonuclease (Rbn)	0	1	0
A1S_3092	putative RND type efflux pump involved in aminoglycoside resistance (adeT)	1	0	0
A1S_3101	toluene tolerance efflux transporter	1	0	0
A1S_3102	toluene tolerance efflux transporter	1	0	0
A1S_3103	toluene tolerance efflux transporter	1	0	0
A1S_3117	Putative tetA efflux pump	1	0	0
A1S_3142	Putative membrane protein	1	0	0
A1S_3146	Multidrug efflux transport protein	1	0	0
A1S_3153	Putative membrane protein	1	0	0
A1S_3154	Putative membrane protein	1	0	0
A1S_3165	pilin like competence factor	0	1	1
A1S_3166	pilin like competence factor	0	1	1
A1S_3167	possible pilus assembly protein tip-associated adhesin PilY1	0	1	0
A1S_3168	Possible pilus assembly protein PilW	0	1	0

A1S_3177	fimbrial protein	0	1	1
A1S_3191	putative outer membrane protein (ComQ)	0	1	0
A1S_3192	Putative lipoprotein (ComL)	0	1	0
A1S_3193	Putative membrane protein (ComO)	0	1	0
A1S_3194	Putative membrane protein (ComN)	0	1	0
A1S_3195	putative membrane protein ComM	0	1	0
A1S_3214	cation efflux system protein	1	0	0
A1S_3217	RND divalent metal cation efflux transporter	1	0	0
A1S_3219	Putative RND family drug transporter	1	0	0
A1S_3220	cation efflux system protein	1	0	0
A1S_3295	Excinuclease ABC subunit A	1	1	0
A1S_3313	3-deoxy-D-manno-2-octulosonate transferase	0	1	0
A1S_3315	putative transcriptional regulator (ArsR family)	0	0	1
A1S_3324	putative ferric siderophore receptor protein	0	1	0
A1S_3325	putative ferric siderophore receptor protein	0	1	0
A1S_3330	UDP-3-O-acyl-N-acetylglucosamine deacetylase	0	1	1
A1S_3339	putative ferric siderophore receptor protein	0	1	0
A1S_3355	hypothetical protein	0	1	0

A1S_3359	Topoisomerase IV subunit B	1	0	0
A1S_3374	Positive pho regulon response regulator	0	1	0
A1S_3375	Positive pho regulon response regulator	0	1	0
A1S_3393	hypothetical protein	0	1	0
A1S_3394	hypothetical protein	0	1	0
A1S_3410	Putative acyltransferase	0	1	0
A1S_3445	Putative RND family cation/multidrug efflux pump	1	0	0
A1S_3446	Putative RND family cation/multidrug efflux pump	1	0	0
A1S_3459	hypothetical protein	0	1	0
A1S_3493	hypothetical protein	0	1	0
A1S_3577	hypothetical protein	0	1	0
A1S_3792	hypothetical protein	0	0	1
A1S_3841	hypothetical protein	0	1	0
A1S_3881	hypothetical protein	0	1	0
A1S_3882	hypothetical protein	0	1	0

Membership was assigned by applying a rule set fixed before testing to STRING v12 pre-computed functional terms (GO biological process and cellular component, UniProt keywords, Pfam domains, and STRING local network clusters) and to RefSeq product names. Genes may belong to more than one set.

Table S12. Cross-species summary of the R/P/B overlap analysis (upper block) and robustness variants for Acinetobacter baumannii ATCC 17978 (lower block). One-sided hypergeometric tests against the species-specific STRING v12 background; q = Benjamini–Hochberg adjusted P.

Order	Strain	Background N	R (n)	P (n)	B (n)	Candidate genes (≥2 sets)	Overlap	Intersection (n)	OR	P	q
Pseudomonadales	P. aeruginosa PAO1	5,571	81	105	73	48	R∩P	18	17.74	3.50e-15	5.25e-15

Pseudomonadales	P. aeruginosa PAO1	5,571	81	105	73	48	P∩B	29	47.02	1.52e-32	4.56e-32
Pseudomonadales	P. aeruginosa PAO1	5,571	81	105	73	48	R∩B	1	0.94	6.59e-01	6.59e-01
Enterobacterales	E. coli K-12 MG1655	4,140	69	106	42	33	R∩P	26	30.16	4.88e-25	1.46e-24
Enterobacterales	E. coli K-12 MG1655	4,140	69	106	42	33	P∩B	7	8.08	7.63e-05	1.14e-04
Enterobacterales	E. coli K-12 MG1655	4,140	69	106	42	33	R∩B	0	0.00	1.00e+00	1.00e+00
Moraxellales	A. baumannii ATCC 17978	3,787	96	213	45	37	R∩P	9	1.77	8.85e-02	1.33e-01
Moraxellales	A. baumannii ATCC 17978	3,787	96	213	45	37	P∩B	28	31.67	8.36e-25	2.51e-24
Moraxellales	A. baumannii ATCC 17978	3,787	96	213	45	37	R∩B	0	0.00	1.00e+00	1.00e+00

Variant (A. baumannii)	R (n)	P (n)	B (n)	Overlap	Intersection (n)	OR	P	q
Pre-specified rules	96	213	45	R∩P	9	1.77	8.85e-02	1.33e-01
Pre-specified rules	96	213	45	P∩B	28	31.67	8.36e-25	2.51e-24
Stringent P set	96	185	45	R∩P	9	2.07	4.32e-02	6.47e-02

Stringent P set	96	185	45	P∩B	27	34.03	4.83e-25	1.45e-24
LPS/O-antigen module removed (n = 49)	92	164	43	R∩P	5	1.28	3.68e-01	5.52e-01
LPS/O-antigen module removed (n = 49)	92	164	43	P∩B	26	39.96	1.09e-25	3.27e-25

The stringent P set removes two broadly defined GO biological-process terms ("Defense response" and "Defense response to other organism") and the prophage/prophage-structural product-name rule, reducing the P set from 213 to 185 genes. The dual-annotation sensitivity removes the LPS/O-antigen modification module from all three sets.

Table S13. Specificity control for the Acinetobacter baumannii ATCC 17978 gene sets. Overlap of the resistance (R), phage receptor/defense (P), and biofilm (B) sets with four unrelated control gene sets of comparable size, tested by the same one-sided hypergeometric test against the same background (N = 3,787). The second block reproduces the observed overlaps for direct comparison.

Control gene set	Control set size (n)	Target set	Target set size (n)	Intersection (n)	OR	P
Central metabolism (glycolysis, TCA cycle, oxidative phosphorylation)	81	R	96	0	0.00	1.000
Central metabolism (glycolysis, TCA cycle, oxidative phosphorylation)	81	P	213	0	0.00	1.000
Central metabolism (glycolysis, TCA cycle, oxidative phosphorylation)	81	B	45	0	0.00	1.000
Ribosome / translation	68	R	96	0	0.00	1.000

machinery						
Ribosome / translation machinery	68	P	213	0	0.00	1.000
Ribosome / translation machinery	68	B	45	0	0.00	1.000
Bacterial secretion system (envelope-proximal)	34	R	96	0	0.00	1.000
Bacterial secretion system (envelope-proximal)	34	P	213	0	0.00	1.000
Bacterial secretion system (envelope-proximal)	34	B	45	0	0.00	1.000
Purine metabolism	57	R	96	0	0.00	1.000
Purine metabolism	57	P	213	0	0.00	1.000
Purine metabolism	57	B	45	0	0.00	1.000

Overlap	|A|	|B|	Intersection (n)	OR	P	q
R ∩ P	96	213	9	1.77	8.849e-02	1.327e-01
P ∩ B	213	45	28	31.67	8.360e-25	2.508e-24
R ∩ B	96	45	0	0.00	1.000e+00	1.000e+00

Control gene sets were retrieved from KEGG for A. baumannii (organism code acb): central metabolism (map00010, map00020, map00190), ribosome (map03010), bacterial secretion system (map03070), and purine metabolism (map00230). None of the twelve control contrasts was significant.
